# Environment-driven control of fungi in subterranean ecosystems: the case of La Garma Cave (northern Spain)

**DOI:** 10.1007/s10123-021-00193-x

**Published:** 2021-07-22

**Authors:** Sergio Sanchez-Moral, Valme Jurado, Angel Fernandez-Cortes, Soledad Cuezva, Tamara Martin-Pozas, Jose Luis Gonzalez-Pimentel, Roberto Ontañon, Cesareo Saiz-Jimenez

**Affiliations:** 1 Museo Nacional de Ciencias Naturales (MNCN-CSIC), 28006 Madrid, Spain; 2 Instituto de Recursos Naturales y Agrobiologia (IRNAS-CSIC), 41012 Sevilla, Spain; 3 Departamento de Biologia y Geologia, Universidad de Almeria, 04120 Almeria, Spain; 4 Departamento de Geologia, Geografia y Ciencias Ambientales, Universidad de Alcala de Henares, 28805 Madrid, Spain; 5 Laboratorio Hercules, Universidade de Evora, 7000-809 Evora, Portugal; 6 Museo de Prehistoria y Arqueologia de Cantabria, 39009 Santander, Spain

**Keywords:** Cave, Paleolithic art, Microclimate, Aerobiology, *Ascomycota*, *Basidiomycota*

## Abstract

Airborne microorganisms can cause important conservation problems in caves with Paleolithic art and therefore the knowledge of cave aerodynamic is essential. La Garma Cave (Cantabria, Spain), an exceptional archaeological site with several levels of galleries interconnected and two entrances, presents a complex atmospheric dynamics. An approach including aerobiological sampling together with microclimate monitoring was applied to assess the factors controlling the origin of airborne fungi. Here we show that winter ventilation is critical for the increasing of *Basidiomycota* spores in the cave air and the highest concentrations were found in the most ventilated areas. On the contrary, *Ascomycota* spores prevailed in absence of ventilation. Besides, most *Ascomycota* were linked to insects and bats that visit or inhabit the cave. The combination of aerobiological and microclimate data constitutes a good approach to evaluate the influence of external climatic conditions and design the most suitable strategies for the conservation of cultural heritage in the cave environment.

**Supplementary Information:**

The online version contains supplementary material available at 10.1007/s10123-021-00193-x.

## Introduction

The identification, protection, and preservation of the cultural heritage of exceptional value for humankind are recommended by the United Nations Educational, Scientific and Cultural Organization (UNESCO). Cultural heritage comprises sites with historical, esthetic, archaeological, scientific, ethnological, or anthropological value, among which are included the caves with Paleolithic paintings.

**Fig. 1:**
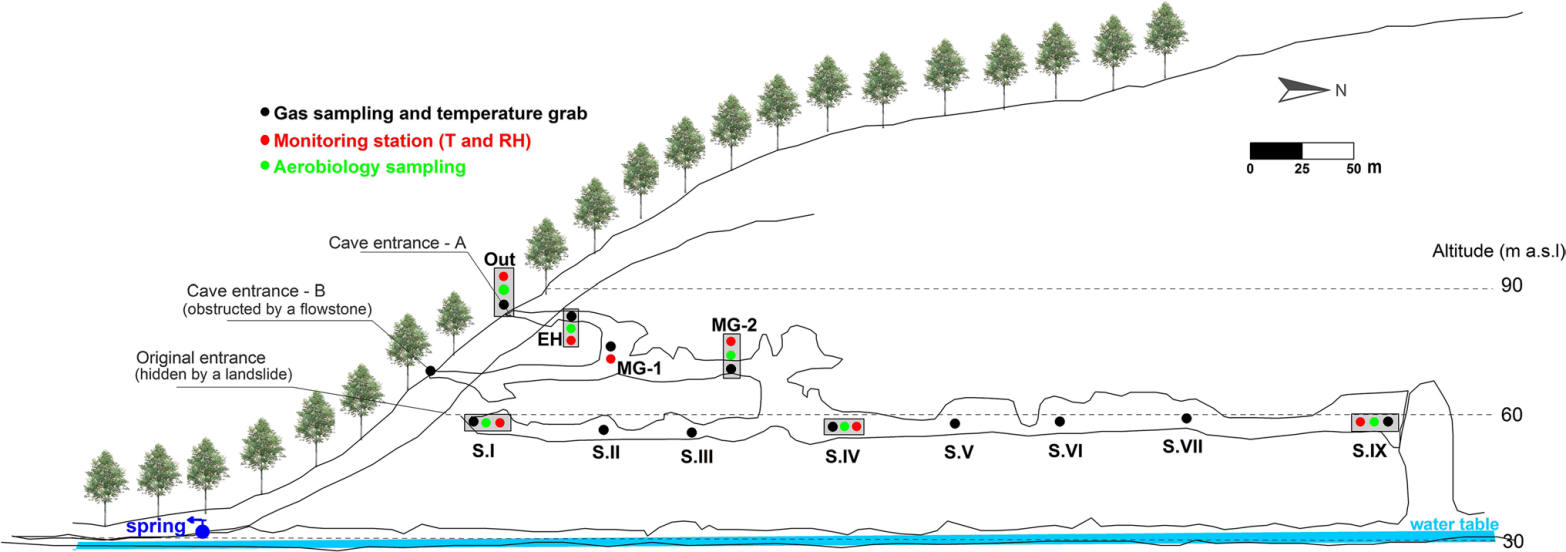
Cross-section of La Garma Cave showing the position of monitoring stations and the location of air and aerobiology sampling points. EH, Entrance Hall; MG, Middle Gallery; S#, numbered sectors of Lower Gallery

Spain is a privileged territory in terms of abundance and variety of subterranean karst forms with cave art. “Cave of Altamira and Paleolithic Cave Art of Northern Spain” is a World Heritage Site which includes La Garma Cave (Fig. 1). The cave contains one of the most important archaeological sites in the world since it collects the evolutionary history of its human population over a long period of time (Arias and Ontañón 2012, 2014).

In the natural state, many caves are oligotrophic and have little or no connection to the external atmosphere. Organic matter in these ecosystems is limited, entering through the main entrance, via infiltration water or introduced by external organisms. Unfortunately, caves usually show a high and fragile environmental stability when compared with epigean habitats that makes it very susceptible to disturbances due to anthropogenic activities. For millennia, the caves have maintained a balance between microorganisms and indigenous cave animals, which is broken when the cave environment is disturbed by human activities (tourist facilities, archaeological activities, agriculture, livestock, etc.) (Saiz-Jimenez 2013). Actually, there exist several examples of how tourism can produce significant environmental impacts caused by the adaptation of caves for visits (Saiz-Jimenez et al. 2011; Lasheras, 2014; Geneste and Mauriac 2014; Alabouvette and Saiz-Jimenez, 2016; Cigna 2016).

Regardless of human activity, the connection degree of the subterranean environments with the outer atmosphere can vary depending on the morphology of the cave and the atmospheric-climatic and edaphic conditions (Garcia-Anton et al. 2017; Pla et al. 2017). There is substantive evidence that subterranean cavities experienced significant daily and seasonal changes in CO_2_ and CH_4_ concentrations (Fernandez-Cortes et al. 2015a, b; Mattey et al. 2013). These variations involve the exchange of large amounts of subsurface air with the lower troposphere and are considered good tracers to study the periods of stagnation, recharge, and storage processes of gases in the subsurface environment (Cuezva et al. 2011; Ojeda et al. 2019).

In caves, a great number of microorganisms enter through the external air and water dripping, occupying all niches. Besides, in caves with Paleolithic art, the air acts as a vehicle for the dispersion of microorganisms involved in the biodeterioration of paintings (Jurado et al. 2009). There is a wide literature on the characterization of subterranean microbiota, especially on bacterial communities (Northup and Lavoie 2001; Fernandez-Cortes et al. 2011; Garcia-Anton et al. 2014). However, references focusing on the influence of bioclimatic parameters on the colonization, dissemination, and ecology of microorganisms in natural subterranean environments are less frequent (Mulec et al. 2017; Wojkowski et al. 2019). Reports on fungi in Spanish caves are relatively scarce compared to other microbiological studies on terrestrial and aquatic ecosystems. However, in the last decade, the studies on caves have increased, both in Spain (Jurado et al. 2010; Fernandez-Cortes et al. 2011; Domínguez-Moñino et al. 2012, 2014; Garcia-Anton et al. 2014; Nováková et al. 2014a,b) and other European countries (Bastian et al. 2009, 2010; Nováková et al. 2012; Martin-Sanchez et al. 2012a,b; Mulec et al. 2020). Of particular interest are the studies aimed at knowing cave microbial ecology as a way to adopt protective measures to prevent microbial outbreaks (Jurado et al. 2010; Porca et al. 2011; Domínguez-Moñino et al. 2014; Martin-Sanchez et al. 2014, 2015; Alonso et al. 2019).

Aerobiological sampling is now being introduced as a cave management tool to control dispersion and fungal colonization on speleothems, sediments, and rocks. The combination of different analytical methods, e.g., linking trace gases, microclimate, and aerobiological data to monitor the entry and dispersion of microorganisms in caves could allow detecting the extent of human-induced changes that, in certain cave areas, can be very hazardous.

The main objective of this aerobiological study is to evaluate the factors controlling fungal distribution into the cave and to be able to elucidate its origin and, when appropriate, establish control measures for preventing the proliferation of undesirable microorganisms. To accomplish this objective, we relied on microclimate monitoring with a double focus. Firstly, an investigation on the microclimate conditions inside and outside the cave to know the meteorological constraints on the cave ventilation and its effect on the gaseous composition of the subterranean atmosphere and, secondly, an analysis of the mechanisms involved in the dynamic of fungal spores. This approach permitted us to know the origin of fungi in the air and to verify the influence of external climatic conditions on the cave atmosphere and mycobiota. This knowledge will serve to design the most appropriate strategies for the conservation of subterranean sites with a valuable heritage.

## Materials and methods

### Study site and geomorphological settings

**Fig. 2:**
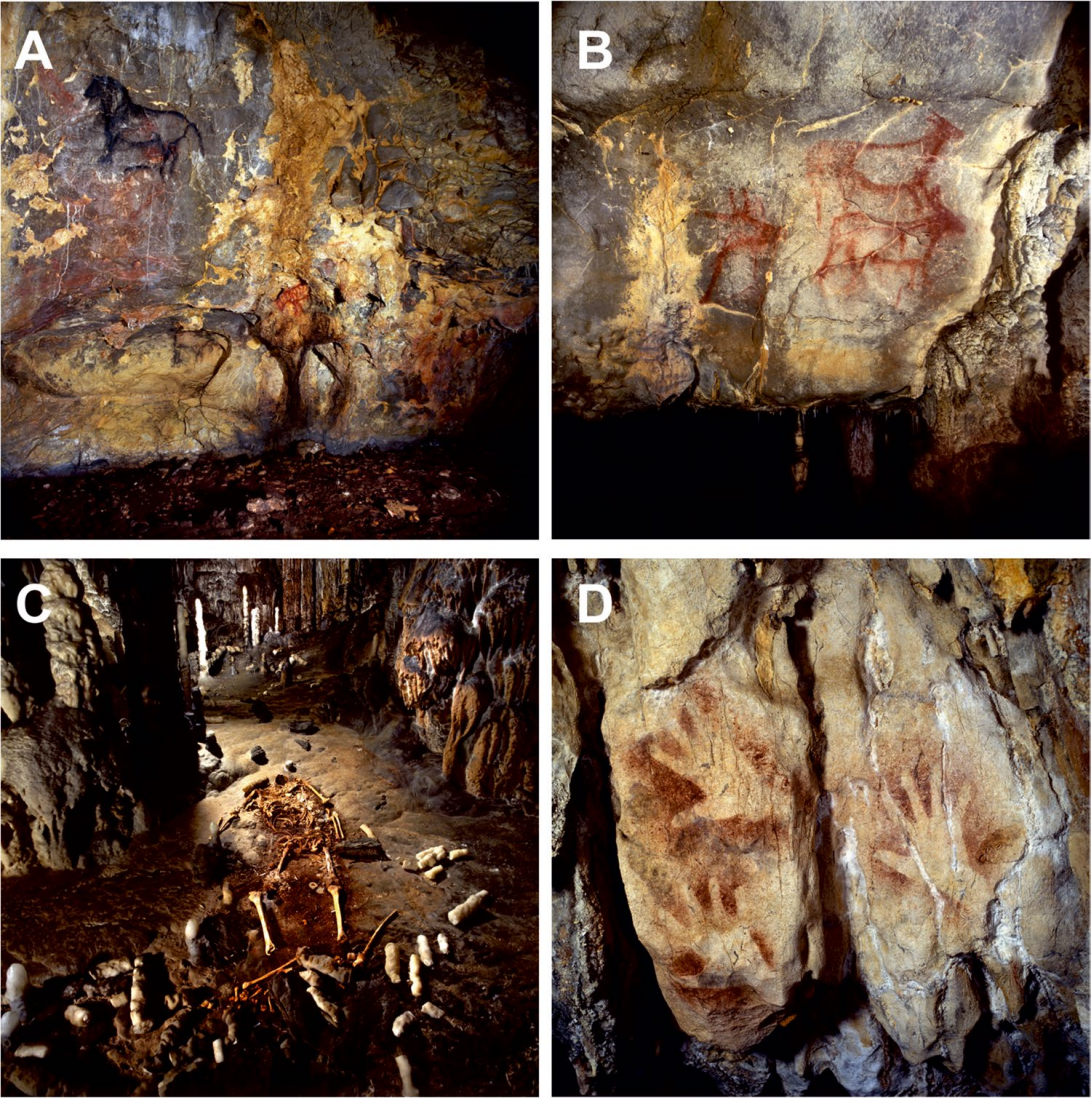
Cave Paleolithic art and archaeological remains on the main sampling sites in La Garma Cave. (**A**) Painting of a horse in Magdalenian style decorating the settlement floor of the same chronology in Sector I; (**B**) Composition of aurochs, ibex, and red deer painted in red from the Early Upper Paleolithic in Sector IV; (**C**) Visigoth burial of a female in Sector V; (**D**) Negative hand stencils painted in red from the Early Upper Paleolithic in Sector IX

La Garma Cave (43.43°N; 3.67°W) was discovered in November 1995 and is part of a complex karst system of galleries located at different heights and connected by small potholes. The cave, located in the town of Omoño, Ribamontán al Monte municipality (Cantabria), opens on the southern slope of La Garma hill (187 m a.s.l.) and is made up of calcareous materials from the Lower Cretaceous (Aptiense-Albiense) in a contact zone between the Ramales Formation (Urgonian Complex) and the Bielva Formation. Soil thickness across La Garma area usually varies between 60 and 150 cm (Comas-Bru and McDermott 2015). The cave area is mainly populated by *Quercus* spp*.* The development of the karstification process in the area was conditioned by the fracturing system of Cretaceous materials that led to a strong dissolution in favor of subvertical fractures with a preferential NNE-SSW direction (Comas-Bru and McDermott 2015; Cuezva et al. 2016). The result was a karst system (Fig. 1) with up to nine known levels of horizontally developed galleries, of which these four are easily accessible:

- Basal Level: coincides with the water table, at a height of 30–35 m from the current valley level. The water circulates and emerges outside through a spring called “Fuente en Cueva”. At present in speleological and archaeological explorations.
- Lower Gallery: developed 60 m above sea level and 30 m above the water table. It presents the longest accessible section and has undergone stages of intense occupation that have left an exceptional archaeological record (Fig. 2), with the particularity of having remained practically intact (Ontañón 2003). This gallery has several sectors (I–IX) and had direct access to the outside, currently blocked by detrital materials probably coming from the sliding of a mass of rock, soil, and sediments in favor of the slope. The dating of this landslide is still pending, although different evidences suggest that this closure occurred about 16,000 years ago.
- Middle Gallery: 10–12 m above the Lower Gallery. In the past, a direct connection to the outside took place through La Garma B, a small cavity separated from the Middle Gallery by the development of large stalagmites and a flowstone blocking the gallery. These formations currently prevent the aerodynamic connection between the exterior and the Middle Gallery through La Garma B entrance.
- La Garma A: 12–13 m above the Middle Gallery. Cave entrance in direct connection with the outside is the current access to the cavity including the Entrance Hall on the same level and giving way to the rest of the levels of the karst system.

La Garma is an outstanding example of long-term human activity in a karstic system (Arias and Ontañón 2012). This hill contains ten archaeological sites, most of them located inside caves, which have revealed evidence of human presence from the Lower Paleolithic to the Middle Ages. These sites are located on the southern side of the hill. The most important are the Lower Gallery and La Garma A. The former is a passage 300-m long where several areas with astounding concentrations of Middle Magdalenian remains (c 16,500 BP) have been found on the floor surface, as well as an important collection of Paleolithic cave art, of varying chronology (Fig. 2). It was also used as a burial cave in the Middle Ages, and further evidence of cave exploration at that period has been found.

La Garma A site has yielded a long stratigraphic sequence, which begins in the Lower Paleolithic, and includes strata of all the main periods of the Upper Paleolithic, the Mesolithic, Neolithic, Chalcolithic, and Bronze Age, in addition to some medieval objects on the ground surface. Between the Lower Gallery and La Garma A, there is another level at 70 m above sea level, in whose entrance (La Garma B) a Chalcolithic and Bronze Age burial deposit has been excavated, and the interior (Middle Gallery) contains some Paleolithic paintings and a Lower Paleolithic deposit, as well as paleontological remains (brown bears). In a deep sector of the Basal Level, other evidence of funerary deposits of the Middle Ages has been found, and in a small gallery between that one and the Lower Gallery, a skeleton of cave lion and tracks of human footprints, whose study is now ongoing. El Truchiro Cave, a small cave located at the same level, has yielded Mesolithic and Chalcolithic human burials. Two sections of cave passage on a higher level than La Garma A, called La Garma C and La Garma D, were the location of Chalcolithic burial deposits. Archaeological sites have been also studied in other parts of the hill, apparently unconnected with the main cave system. These are Peredo Cave, with Bronze Age burials, El Valladar Cave, with medieval remains, and Cueva del Mar, with a badly deteriorated deposit that includes Mesolithic levels.

### Microclimatology

Outdoor weather conditions were recorded every half hour with a HOBO HWS weather station (Onset Computer Corporation, USA) from March 2015 to June 2016. This station is equipped with a 12-bit smart sensor (Onset Computer Corporation model S-THB) to measure air temperature and relative humidity. Accuracy is ± 0.2 °C from 0 to 50 °C for air temperature, and ± 2.5% from 10 to 90% relative humidity, up to a maximum of ± 3.5% (above 95%), for relative humidity. The station also has a tipping-bucket rain gauge (S-RGB model) for rainfall measurements (measurement range: 0–12.7 cm, resolution: 0.2 mm) and a Davis® smart wind speed and direction sensor for measuring wind and gust speeds (measurement range: 0–76 m/s, accuracy: ± 1.1 m/s, resolution: 0.5 m/s). A set of autonomous temperature and relative humidity recorders was installed inside the karst system (Tinytag TGP-4500 with a resolution < 0.01 °C), paying special attention to the geomorphology of galleries and the distance to the exterior (Fig. 1). Hence, stations were placed in three points of the Lower Gallery (Sectors I, IV, and IX), two in the Middle Gallery, and one in the Upper Gallery. Moreover, a multiparametric and spatially resolved monitoring of the main air parameters: temperature (5611 T, Hart Scientific) and relative humidity, Hygropalm 1 (Rotronic) was conducted seasonally in tandem with cave air sampling with micro-diaphragm gas pumps (KNF Neuberger) in a pre-established network of 11 points (Fig. 1), covering the Upper, Middle, and Lower Galleries. Air samples were stored in 1-L RITTER air/gas sampling bags specifically designed to ensure that the collected gases remain uncontaminated and unaltered. Bag samples were analyzed within 48 h of sampling for methane (CH_4_) concentration by a laser-based analyzer (Picarro G2201-i, California, USA). The concentration of CH_4_ in the cave atmosphere depends on the rate of exchange of air with the exterior and is a good indicator of the ventilation rates of the karst system, both on a temporal and spatial scale (Fernandez-Cortes et al. 2015b; Webster et al. 2018).

### Aerobiological sampling

Four sampling campaigns were carried out on March 24, 2015, September 17, 2015, November 19, 2015, and February 17, 2016. In each campaign, a total of five points inside the cave and one outside were sampled. They covered the different cave galleries and were labeled as Entrance Hall, Middle Gallery, Sector I, Sector IV, and Sector IX.

The sampling was carried out with a Duo SAS (Surface Air System) Super 360 system (International PBI, Milan, Italy). This equipment is a type of suction impact collector, which has been widely used in aerobiological studies. It allows the detection of a great diversity of culturable microorganisms by filtering a preselected volume of air through two heads provided with a series of holes.

Two replicates of air samples (100 L each one) were directly aspirated, using a 219-hole impactor, onto the appropriate culture media contained in 90-mm Petri dishes. The culture medium used to promote the growth of fungi was Dichloran Rose-Bengal Chloramphenicol Agar (Merck, Darmstad, Germany).

The volumes of air samples were based on a previous study (Porca et al. 2011). Volumes higher than 100 L resulted in the growth of an excessive number of colonies on the culture medium to be efficiently counted.

The final amounts of fungi in each air sample were expressed as colony-forming units per cubic meter following Duo SAS sampler manufacturer’s instructions. The number of colonies counted on the surface of the culture plates was corrected for the statistical possibility of multiples particles passing through the same hole.

Viable counts and isolations in pure culture were independently performed for each colony type with different morphological characters, and each replicate of the air sample. The fungi were isolated on malt extract agar medium (MEA), including 50 mg/L chloramphenicol which inhibits a wide variety of bacteria and subsequently stored at 4 °C.

Fungal mycelia were collected from the plates and transferred to a 1.5-mL Eppendorf tube containing 500 µL TNE buffer (10 mM Tris–HCl, 100 mM NaCl, 1 mM EDTA; pH 8) and glass beads. The mixture was shaken in a cell disrupter (Fast Prep-24, Solon, USA) at full speed for 3 min. The DNA was purified by phenol/chloroform extraction and ethanol precipitation (Dominguez-Moñino et al. 2021).

The fungal internal transcribed spacer (ITS) regions, including ITS1, 5.8S rDNA, and ITS2, were amplified using the primers ITS1 5′-TCCGTAGGTGAACCTGCGG-3′ and ITS4 5′-TCCTCCGCTTATTGATATGC-3′ (White et al. 1990). PCR reactions were performed in 50-μL volumes, containing 5 μL of 10 × PCR buffer, 2 μL of 50 mM MgCl2, 5 μL of 2 mM dNTP mix (Invitrogen, Carlsbad, CA, USA), 0.5 μL of 50 μM of each primer (Macrogen, Seoul, Korea), 10–20 ng of the extracted DNA as template, 1.25 units of Taq DNA polymerase (Bioline, GC Biotech, The Netherlands), and the rest of volume of sterile ultrapure water. Reactions were performed in duplicate, and negative controls (containing no DNA) were included in each PCR trial. PCR amplifications were performed in a BioRad iCycler thermal cycler (BioRad, Hercules, CA, USA) using the following cycling parameters: 2 min of initial denaturing step at 95 °C, followed by 35 cycles of denaturing (95 °C for 1 min), annealing (55 °C for 1 min), and extension (72 °C for 2 min), with an additional extension step at 72 °C for 10 min at the end. To evaluate the PCR results, all products were electrophoresed on 1% (w/v) agarose gels, stained with SYBR Green I (Roche Diagnostics, Mannheim, Germany), and visualized under UV light.

Positive PCR products were sent to Macrogen Inc. (Amsterdam, The Netherlands) for sequencing using the same primer set. In order to approximate the phylogenetic identification of strains, the received sequences were compared, using BLASTn algorithm, to the non-redundant databases of sequences deposited at the National Center for Biotechnology Information (NCBI). The sequences were deposited into the GenBank database under accession numbers MW826125-MW826214.

## Results

### Outdoor climate

La Garma Cave is located in a temperate climate zone (Comas-Bru and McDermott 2015). Mean annual temperature recorded in the years 2015 and 2016 was 13.92 and 13.98 °C, respectively. Mean temperature of the coldest months ranged between 7.26 °C (February 2015) and 8.65 °C (December 2016) and the mean temperature of the warmest month was around 20.2 °C (July–August). From April to November, the mean temperature is above 10 °C. The total annual precipitation recorded in the 2015–2016 biennium slightly exceeds 1000 mm (1084 and 1024 mm in 2015 and 2016, respectively). The rainiest months are repeated in February and November of each year, with relative maximums varying from 120–172 mm (November) to 236–245 mm (February). During the period studied, rainfall has been recorded in the form of rain in all months, the driest months (20–30 mm/month) varying from 1 year to the next; April, June, and December (2015) or August (2016).

The prevailing wind regime in the surroundings of La Garma Cave largely controls local meteorological phenomena and, in particular, sudden changes in air temperature and rainfall events. The lower intensity winds (speed less than 10 k/h) are the most frequent throughout the year and usually have a north component. The wind of greater intensity (> 20 km/h) is less frequent and corresponds preferably to a south-southwest component wind coming from more inland areas, i.e., farther away from the coastline, which brings warmer air. A remarkable fact is the absence of rainfall associated with the record of south winds. This indicates a clear influence of Foehn effect on the rainfall regime at a regional scale and due to the presence of the Cantabrian mountain range, south of the study area. The xeric conditions associated with the south wind in the surroundings of La Garma Cave usually occur between August and October and are related to cyclonic movements on a regional scale that lead to intense wind speeds that, as we will see later, are directly reflected in the climatic conditions of the cavity.

### Subterranean climate

**Fig. 3:**
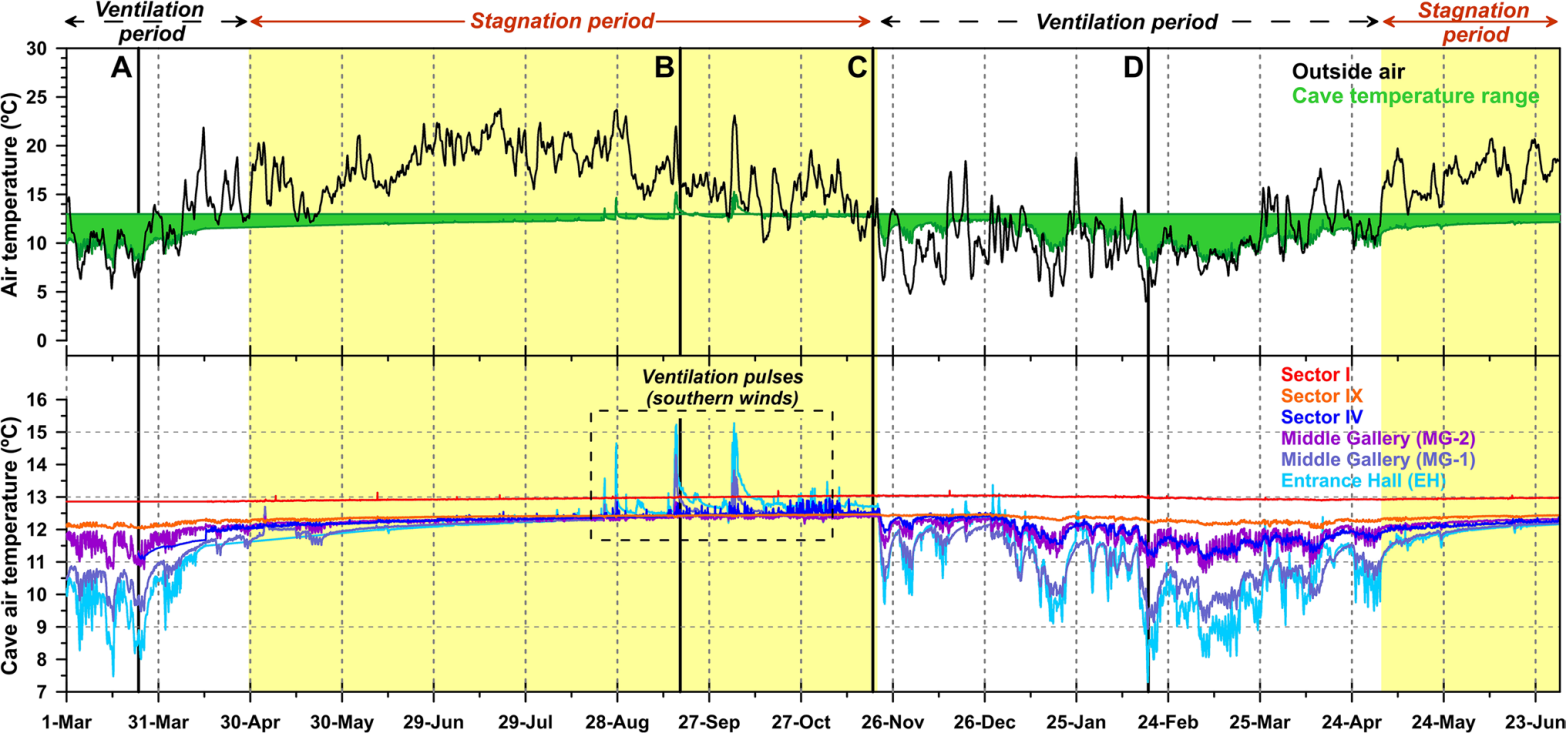
Evolution of the outdoor air temperature and in the six environmental monitoring points of La Garma Cave (March 2015–June 2016). (A) to (D) locate the aerobiological samplings. The location for each temperature series is referred to Fig. 1

The characteristics and seasonal evolution of temperature in a karst system determine the degree of connection between the exterior and the subterranean environment as well as the concentration of gases and particles in the cave atmosphere. The continuous thermohygrometric recording carried out outdoor and in the six indoor stations (Fig. 3 and Table 1) allows us to know the spatiotemporal patterns and distribution of temperature in the different areas and levels of the karst system.

**Table 1:** Statistical data of temperature (°C) in the different monitored points in La Garma Cave during period 2015–2016, based on time series from the monitoring stations (Fig. 1)

Control station	T mean (°C)	T min (°C)	T max (°C)	Mean daily oscillation T (°C)	Anual variation (°C)
Exterior	13.99	− 0.96	34.76	7.86	35.72
Entrance Hall	11.49	7.30	14.42	0.44	7.12
Middle Gallery-1	11.60	8.94	12.75	0.19	3.81
Middle Gallery-2	12.08	1.67	12.49	0.24	1.82
Lower Gallery Sector IV	12.09	1.06	12.73	0.11	1.67
Lower Gallery Sector IX	12.39	12.03	12.54	0.04	0.51
Lower Gallery Sector I	12.96	12.86	13.05	0.02	0.19

The annual mean temperature of the external atmosphere is higher than that of the cave at all monitoring points (Table 1). Besides, the external temperature remains clearly above that of the cave air for 7–8 months of the year (April–May to October–November) and only during 4 months the opposite thermal situation occurs (outdoor T < indoor T, November–December to February–March, Fig. 3). The external-internal temperature difference is much greater during the summer than in the winter stage.

The general thermal pattern of the monitored areas shows that, for most of the year, the internal areas of the Upper and Middle Galleries show lower temperatures (mean, minimum, and maximum) than those of the Lower Gallery (Table 1). This thermal distribution pattern implies (1) a phenomenon of thermal inversion prevails that causes certain aerodynamic instability between galleries (less dense hot air located at lower levels and denser cold air at upper levels), and (2) the Upper and Middle Galleries and Sector IV of Lower Gallery are clearly affected by the entry and circulation of cold air during the winter season (November–December to February –March, ventilation period, Fig. 3).

Since April, the external temperature becomes higher than the cave temperature, and the atmosphere-cave air exchange is drastically reduced (stagnation period, Fig. 3). This thermal pattern causes an air temperature homogenization at the different levels that tends toward a prevailing motion-less atmosphere during summer, with greater interzones thermal stability. So, in the summer period, there is a greater spatial uniformity of the air temperature along the Middle and the Lower Galleries, with temperature varying less than 0.15 °C between sectors. On the contrary, the cave galleries covering Sectors I to III keep hosting during summer an air mass with a higher temperature compared to the rest of the neighboring sectors (+ 0.5 °C, approximately). The general pattern of thermal stability is altered from August to October when a greater thermal gradient between galleries is registered. This period matches with high-intensity south winds that cause a higher frequency of ventilation pulses due to the inlet of warmer air along fissures and bedrock discontinuities linked to cave galleries below the cave entrance — La Garma A. This disruption in the thermal stability is evidenced by an increase in air temperature in the Upper Gallery and Middle Gallery-1 above the temperature recorded in other cave locations (ventilation pulses, Fig. 3).

Inside the cave, the area with the greatest range of thermal oscillation (Entrance Hall) is that closest to the current Entrance — La Garma A (Table 1). Daily and annual thermal oscillation values (Table 1) in the internal zone of the Upper Gallery are high, 0.44 °C and 7.12 °C respectively. Values progressively descend toward the interior of the cavity until reaching its greatest stability in Sector I of the Lower Gallery, where the mean daily oscillation is only 0.02 °C and the annual thermal amplitude of 0.19 °C. Sector IV of the Lower Gallery presents thermal oscillation values ​very similar to those of the innermost zone of the Middle Gallery (Middle Gallery-2), which indicates that both zones have an aerodynamic communication.

**Fig. 4:**
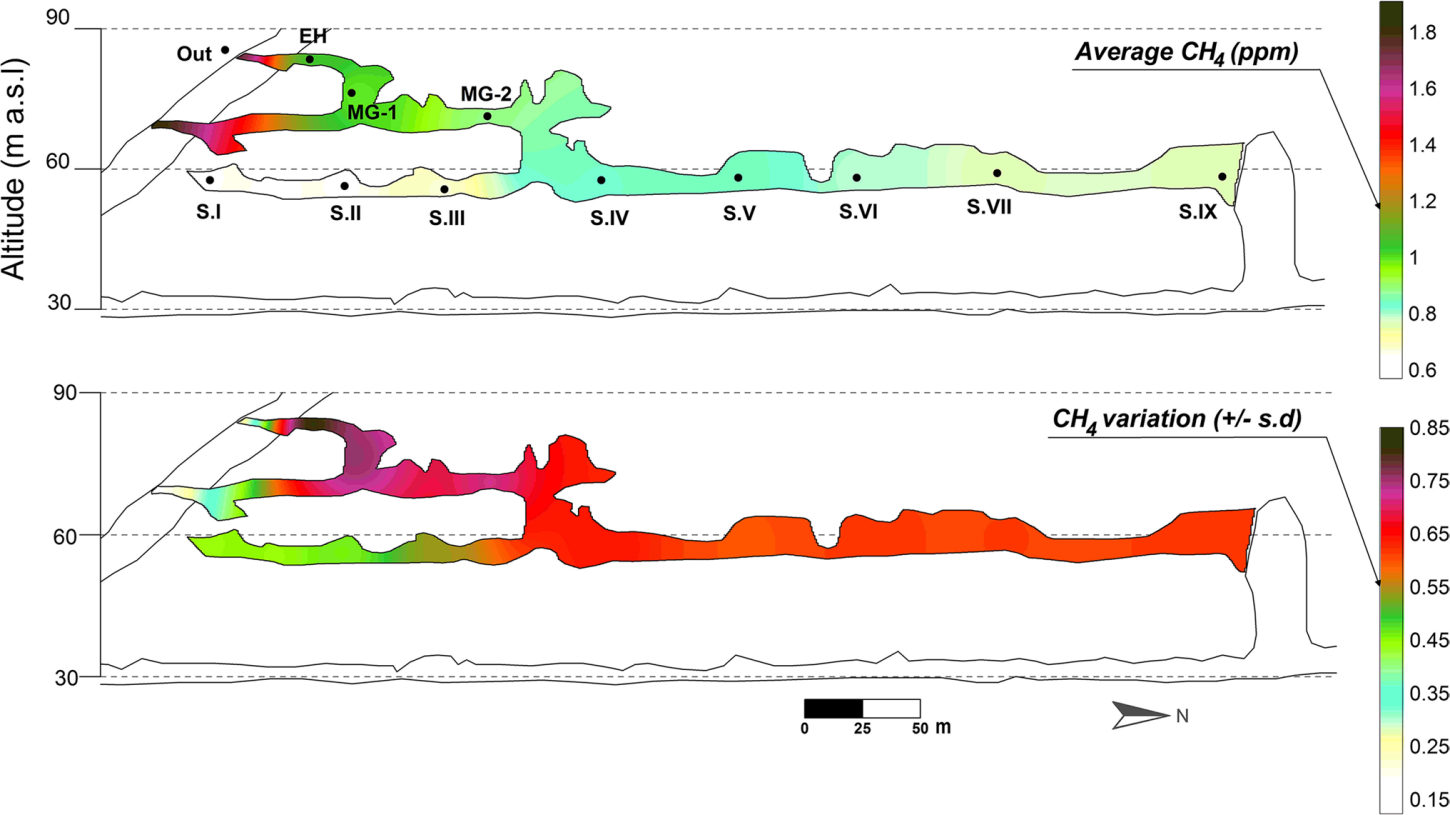
Spatial distribution of the concentration and degree of temporal variation of CH_4_ (March 2015–July 2016). Greater ± s.d. indicates a greater variation/oscillation over time

The blinded end of the Lower Gallery (Sector I) was originally connected with the exterior but a landslide blocked it with detrital materials. This area is, therefore, closer to the surface than other sectors of the Lower Gallery since the thickness of the rock/sediments is less than 2 m. However, the deposit of sedimentary materials that plug the old entrance has a considerable insulating effect. This provokes greater thermal stability in Sectors I and II (Fig. 4), with very low ranges of thermal oscillation (0.19 °C/year and a daily mean of ± 0.02 °C in Sector I, Table 1) and a mean temperature (12.96 °C) higher than the rest and roughly 1 °C lower than the mean temperature at exterior. Consequently, Sectors I and II would be considered as low energy zones from a thermal point of view. Conversely, the largest annual thermal oscillation in the Lower Gallery is recorded in Sectors III and IV as a consequence of air intake processes from the upper levels through the connection area with the Middle Gallery.

### Methane concentration

Methane is a selective tracer for external air entering any dynamically ventilated cave, since subterranean air is firstly enriched in CH_4_ by ventilation pulses and then it tends to be CH_4_-depleted by metanotrophophic activity of bacteria, particularly in locations with stagnated air masses (Fernandez-Cortes et al. 2015b; Waring et al. 2017; Webster et al. 2018). Mean CH_4_ concentration registered during the air samplings carried out inside the cavity was 0.80 ppm, with minimum values reached in the stagnation period covering summer and autumn months and maximum values during winter (Table 2, Fig. 4). The mean concentration of CH_4_ inside the cave (0.80 ppm) is below the mean outside (1.85 ppm) throughout the year, which denotes a remarkable sink effect of this gas. In cave areas with high air renewal, CH_4_ levels are close to those of the outdoor atmosphere (1.8–2.0 ppm), while in the more isolated areas, particularly in the stagnation stages, they fall below these values ​and can reach even zero (July and October 2015, Table 2).

**Table 2:** Methane (CH_4_) concentration in the air from La Garma Cave (February 2015–May 2016). *EH*, Entrance Hall; *MG*, Middle Gallery; *S#*, numbered sectors of Lower Gallery

**Date**	**Cave**												**Outside**
	**EH**	**MG-1**	**MG-2**	**S.I**	**S.II**	**S.III**	**S.IV**	**S.V**	**S.VI**	**S.VII**	**S.IX**	**Mean**	
Feb.-15	1.89	1.72	1.56	1.18	1.23	1.41	1.51	1.47	1.45	1.41	1.41	**1.48**	1.91
Jun.-15	0.89	1.19	0.91	0.54	0.47	0.51	0.75	0.84	0.63	0.64	0.34	**0.70**	1.78
Jul.-15	0.19	0.11	0.14	0.15	0.12	0.09	0.12	0.09	0.12	0.10	0.13	**0.12**	1.55
Oct.-15	0.06	0.05	0.04	0.09	0.13	0.08	0.04	0.21	0.07	0.05	0.07	**0.08**	2.00
Dec.-15	1.91	1.80	1.72	1.13	1.13	1.26	1.64	1.60	1.57	1.53	1.55	**1.53**	1.93
Feb.-16	1.92	1.55	1.54	0.89	0.89	0.90	1.26	1.17	1.21	1.16	1.19	**1.24**	1.94
May-16	0.30	0.40	0.30	0.47	0.48	0.39	0.54	0.54	0.51	0.41	0.59	**0.45**	-
Mean	1.02	0.97	0.89	0.64	0.64	0.66	0.84	0.85	0.79	0.76	0.75	**0.80**	1.85

During the winter season (i.e., air sampling conducted in February and December 2015 and February 2016), the areas closest to the cave Entrance A (namely, Entrance Hall and Middle Gallery-1 and 2) show higher CH_4_ values ​(> 1.3 ppm) as a consequence of the direct entry of external cold and CH_4_-enriched air. The spatial pattern of the CH_4_-depletion enables to identify the connection of the Middle Gallery with the Lower Gallery (Sectors IV–V) as the cave area where the average concentration of CH_4_ decreases significantly (Table 2 and Fig. 4).

The spatial distribution for the standard deviation of CH_4_ also highlights the different air renewal rates of cave location. The largest temporal variability in the concentration of CH_4_ (standard deviation >  ± 0.7 ppm) was primarily registered in the connection area between the Upper and the Middle Galleries. These time oscillations spread through the Middle Gallery and, to a lesser degree, affect the central Sector IV of the Lower Gallery (Fig. 4). This pattern of CH_4_ variability is the result of the convective air circulation that is established between these cave locations and the local atmosphere outdoor. In the one hand, the sequence of external air inlet pulses provokes a mix of atmospheric CH_4_ (1.85 ppm, on average) with the residual CH_4_ of cave air and its subsequent consumption during the stagnation period in which the convective air circulation stops. On the other hand, the slight temporal oscillations in the concentration of CH_4_ in the most isolated areas, i.e., those farthest from the current open cave entrance (Sectors I, II, and IX), confirm that the mixing process with the external atmosphere by advection is almost null there (Fig. 4).

### Aerobiology

In this study, analysis of air samples allowed achieving a representative inventory of the spores of cultivable fungi present in La Garma Cave. However, this culture-dependent approach can afford some lack of diversity due to the culture conditions. We used a nutrient medium incubated at 25 °C for a limited time; such conditions could have favored certain fungi against another; however, the method resulted useful for recovering a significant fungal diversity and estimating their abundance and evolution along the year.

The four aerobiological samplings cover the main aerodynamic periods in the cave (Fig. 3): ventilation period (March 2015 and February 2016), stagnation period with ventilation pulses (September 2015), and stagnation period (November 2015). The concentration of fungal spores outdoor was relatively high during spring and autumn campaigns. In the exterior air, the fungal concentration increases twice in March 2015 compared to November, 2015 (Table 3). Table 3 displays the rates of fungal spores inside the cave, ranging from 10 CFU m^−3^ (November 2015, Sector IX) to 5420 CFU m^−3^ (March 2015, Middle Gallery). In the exterior air, the rates ranged from 2280 CFU m^−3^ in September 2015 to 150 CFU m^−3^ in February 2016. Generally, the higher concentration of spores in the cave air was obtained in March 2015 (ventilation period and spring) and the lower in November 2015 (end of stagnation period with high thermal stability). A comparison of data in Table 3 indicates that in the stagnation period the cave showed the lowest spore concentration in all the galleries and sectors and in the ventilation period the highest. In fact, in the Middle Gallery, the spore concentration increases 180 times in the ventilation versus stagnation periods, and 88 times in Sector IV.

**Table 3:** Rate of fungi (CFU/m^3^) in La Garma Cave and outside

Air sampling	Ventilation period (March 24, 2015)	Stagnation period with ventilation pulses (September 17, 2015)	Stagnation period (November 19, 2015)	Ventilation period (February 17, 2016)
Entrance Hall	N.D	810 (40)	N.D	1010 (80)
Middle Gallery	5420 (30)*	230 (40)	30 (0)	310 (40)
Sector IX	640 (40)	40 (10)	10 (0)	420 (40)
Sector IV	2650 (80)	130 (30)	30 (10)	380 (30)
Sector I	390 (10)	380 (60)	110 (20)	200 (10)
Outside	1520 (40)	2280 (150)	780 (80)	150 (40)

^*^Standard deviation in brackets

**Fig. 5:**
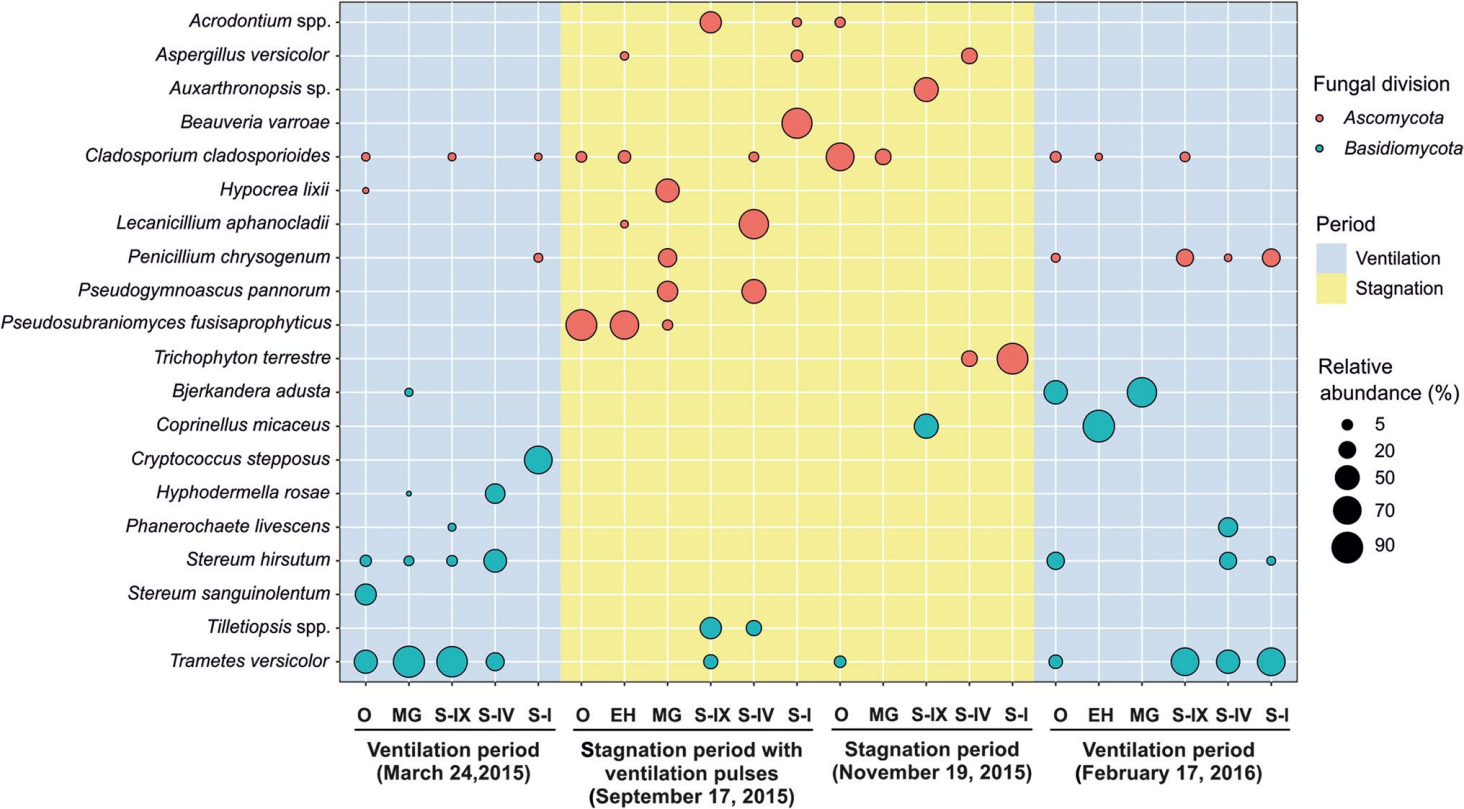
Bubble plot. Most abundant fungi, as distributed in each sampling point and season (O, outdoor air; EH, Entrance Hall; MG, Middle Gallery; S#, numbered sectors of Lower Gallery)

The bubble plot (Fig. 5) illustrates the dispersal of the most abundant fungi across the cave in each period. This figure shows the existence of two main patterns. One in which the dispersal of *Basidiomycota* is mainly concentrated in the ventilation periods (*Trametes versicolor*, *Stereum hirsutum*, *Hyphodermella rosae*, *Bjerkandera adusta*, *Phanerochaete livescens*, *Coprinellus micaceus*), and a second pattern showing the distribution of *Ascomycota* in stagnation periods (*Pseudosubramaniomyces fusisaprophyticus*, *Hypocrea lixii*, *Lecanicillium aphanocladii*, *Beauveria varroae*, *Trichophyton terrestre*, *Cladosporium cladosporioides*). Additionally, we also observed as, in September and November, *Ascomycota* proliferated outdoor and throughout the cave, while in February and March, they were replaced by *Basidiomycota*.

Supplementary Tables 1 to 4 show the abundance of all the fungal species in the different sampling sites and campaigns.

## Discussion

A review on the niches of *Basidiomycota* and *Ascomycota* reported in this work suggests that the abundance of fungal spores in the air of La Garma Cave is governed by two main factors: the ventilation rate and the seasonal release of spores outdoor by specific fungal groups. Besides, fungal origin and CFU m^−3^ concentrations are coherent with the vegetation and abundance of trees in the area surrounding the cave and the wooden logs stored outdoor.

### Ventilation period

**Fig. 6:**
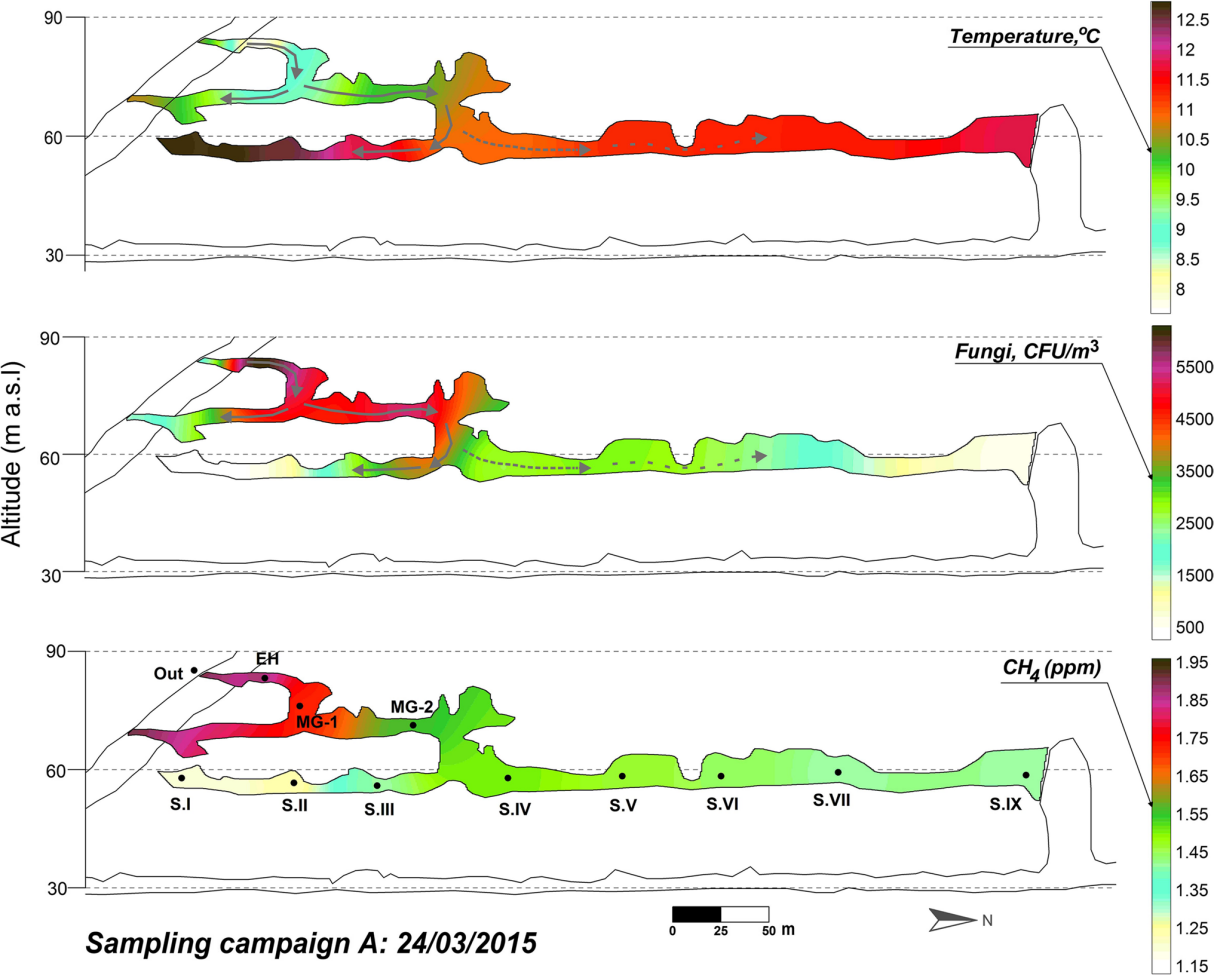
Spatial distribution of the fungal spore concentration, air temperature, and CH_4_ content across La Garma Cave in March 2015

**Fig. 7:**
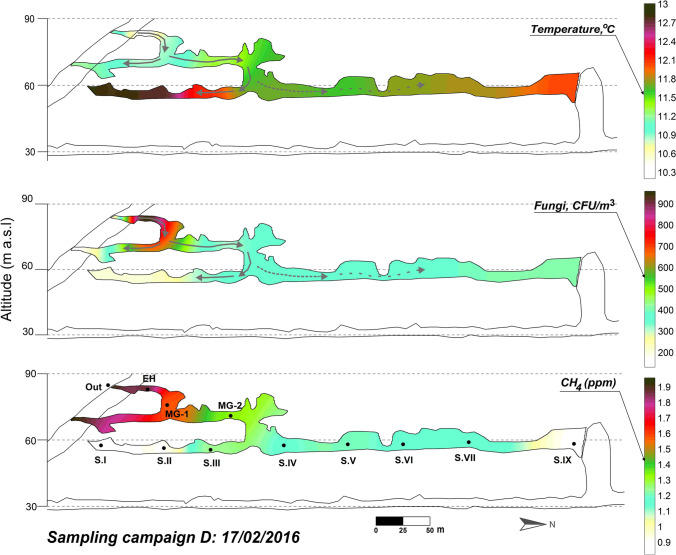
Spatial distribution of the fungal spore concentration, air temperature, and CH_4_ content across La Garma Cave in February 2016

The ventilation period in the cave (December–May) is characterized by the exchange of air between the external atmosphere and the cave through advective air movement. The exterior air, colder and denser than the cave air, enters the cavity through the upper levels and progresses to the Lower Gallery, first intensely affecting Sector IV and then progressively advances toward the rest of that cave levels including Sector IX (Figs. 6 and 7, March 2015 and February 2016, respectively). The result is a notable drop in the air temperature (particularly in Sector IV) with an intense process of renewal of the subterranean air (increase in CH_4_ concentration) and the highest concentrations of fungal spores at each of the sampling points.

Remarkable is the abundance of *T. versicolor* and *S. hirsutum*, in the outdoor air in both ventilation surveys, as well as inside (Fig. 5). In March 2015, it can be observed the progression of their spores from outdoor to the cave interior, but not toward Sector I, which is the most isolated, far from the entrance. In February 2016, we also observed the progression of *T. versicolor* and *S. hirsutum* into the cave interior even reaching Sector I.

These fungi present a worldwide distribution in North Spain, growing on the trunks of dead trees and logs, without showing a preference for a specific species. The release of spores is coincident with their presence in the cave (Welden 1971; Telleria et al. 2010; Justo and Hibbett 2011; Motato-Vásquez et al. 2020). The presence of these *Basidiomycota* inside the cave, therefore, was clearly associated with the entrance of outdoor air and constitutes a great example of how external environmental conditions (and the presence of forest areas) can influence the microbiota of shallow caves. Conversely, in regions without forest (e.g., Nerja Cave in the coastal line of South Spain), *Ascomycota* were the dominant fungi in the cave (Docampo et al. 2011).

In March 2015, in the most isolated sectors, we found a high abundance of the basidiomycetous yeast *Cryptococcus stepposus* and lower of *Naganishia diffluens* (formely *Cryptococcus diffluens*), as well as two *Ascomycota*, *Cladosporium cladosporioides* and *Penicillium chrysogenum*, with low relative abundances. The presence of the genus *Cryptococcus* in caves was related to the frequentation by people and animals (Vaughan-Martini et al. 2000). *Cryptococcus* species were previously isolated from the air of Lascaux Cave (Martin-Sanchez et al. 2014), and bat guano in Japanese caves (Sugita et al. 2005). Therefore, the presence of *Cryptococcus* in the most isolated sector was probably due to the presence of cave animals in this area.

Conversely, *C. cladosporioides* and *P. chrysogenum* also appeared in other periods and cave areas. *C. cladosporioides* was represented the whole year, with their maximum release in November 2015. Interestingly, during this stagnation period, *C. cladosporioides* was not present in the Lower Gallery and, however, it can be observed in the lower sectors during the ventilation seasons, as well associated with periods of southern winds. The genus *Cladosporium* and, specifically, the species *C. cladosporioides* were previously identified in the air of caves (Nováková 2009; Porca et al. 2011; Docampo et al. 2011; Fernandez-Cortes et al. 2011; Garcia-Anton et al. 2014), and on cave mineral substrata (Jurado et al. 2009; Nováková 2009). Remarkably, the spores of *Cladosporium* represented 80% of the total abundance in the air of Altamira Cave. Garcia-Anton et al. (2014) showed that these spores had a clear external origin, reproducing that data obtained for La Garma Cave; therefore, the presence of this fungus in the cave is due to transport from the exterior through air currents.

Spores of the genus *Penicillium* have been found in all samples and practically all sectors, although generally with little abundance over the total number of fungi. In March 2015, species of *P. minioluteum*, *P. digitatum*, *P. diversum*, and *P. chrysogenum* appeared (Supplementary Table 1). In September 2015 (Supplementary Table 2), *P. hispanicum* and *P. thomii* were present in the Entrance Hall, with abundances not exceeding 2%, but in the Middle Gallery, *P. chrysogenum* reached a percentage of 26%, together with *P. brevicompactum* (7%) and *P. westilingii* (5%). In November 2015, *P. roquefortii* represented 5% in Sector I (Supplementary Table 3). The most outstanding *Penicillium* species in La Garma Cave was *P. chrysogenum* (Supplementary Tables 2 and 4). *P. chrysogenum* appeared occasionally inside the cave during March and September, but thrived outside and in the three sectors of the Lower Gallery in February 2016. The genus *Penicillium* is widely distributed in caves (Nováková 2009; Docampo et al. 2011; Fernandez-Cortes et al. 2011; Domínguez-Moñino 2015), and is very common in outdoor and indoor air (Visagie et al. 2014). In February 2016, *Penicillium* species were present in all sectors, with variable abundances, from 24% for *P. chrysogenum* in Sector I, and 21% in Sector IX, to 10% for *P. brevicompactum* in the Middle Gallery and lower percentages for *P. vancouverense*, *P. aurantiovirens*, *P*. *expansum*, and *P. miczynskii* in different sectors (Supplementary Table 4).

Other fungi detected inside the cave in March 2015 were *Bjerkandera adusta* and *Hyphodermella rosae*; the first one was also detected with higher abundance in February 2016, but *H. rosae* was only detected in March 2015. *Bjerkandera adusta*, a polyporaceous basidiomycete, was present in the air of the Middle Gallery in February 2015 and March 2016, and outdoor in this last month, which points to an external origin. This fungus was also isolated from the air of a Slovakian cave (Ogórek 2018). The genus *Hyphodermella* includes white-rot corticioid fungi, widely distributed in Spain. *H. rosae* grows on wood and particularly on *Quercus ilex* in Cantabria, and on *Fagus*, *Alnus*, *Rosmarinus*, etc. (Telleria et al. 2010). Abrego et al. (2014) reported that *H. rosae* was one of the most abundant wood-inhabiting fungi in North Spain forests. According to previous works, the presence of this fungus in well-ventilated areas as Middle Gallery and Sector IV probably indicated an external origin even though it was not detected in the outdoor air sampling.

Differing from the wood degraders, the cosmopolitan *Coprinellus micaceus* thrive on dung, manure, or soils rich in organic matter. *Coprinellus micaceus* is abundantly present in the Entrance Hall (February 2016, Fig. 5). The abundance in the Entrance Hall can be related to their growth on the abundant animal excrements found in this site. A report on the presence of *Coprinus micaceus*, now reclassified as *Coprinellus micaceus*, growing on the mud of Mammoth Cave, USA, dated from the XIX century (Call, 1897). Species of the genus *Coprinellus* have also been recorded in caves from Spain (Docampo et al. 2011), Germany (Andersson 2004), Mexico (Hoffmann et al. 1986), and the USA (Barr 1968; Zhang et al. 2014), among others.

### Air stagnation period

**Fig. 8:**
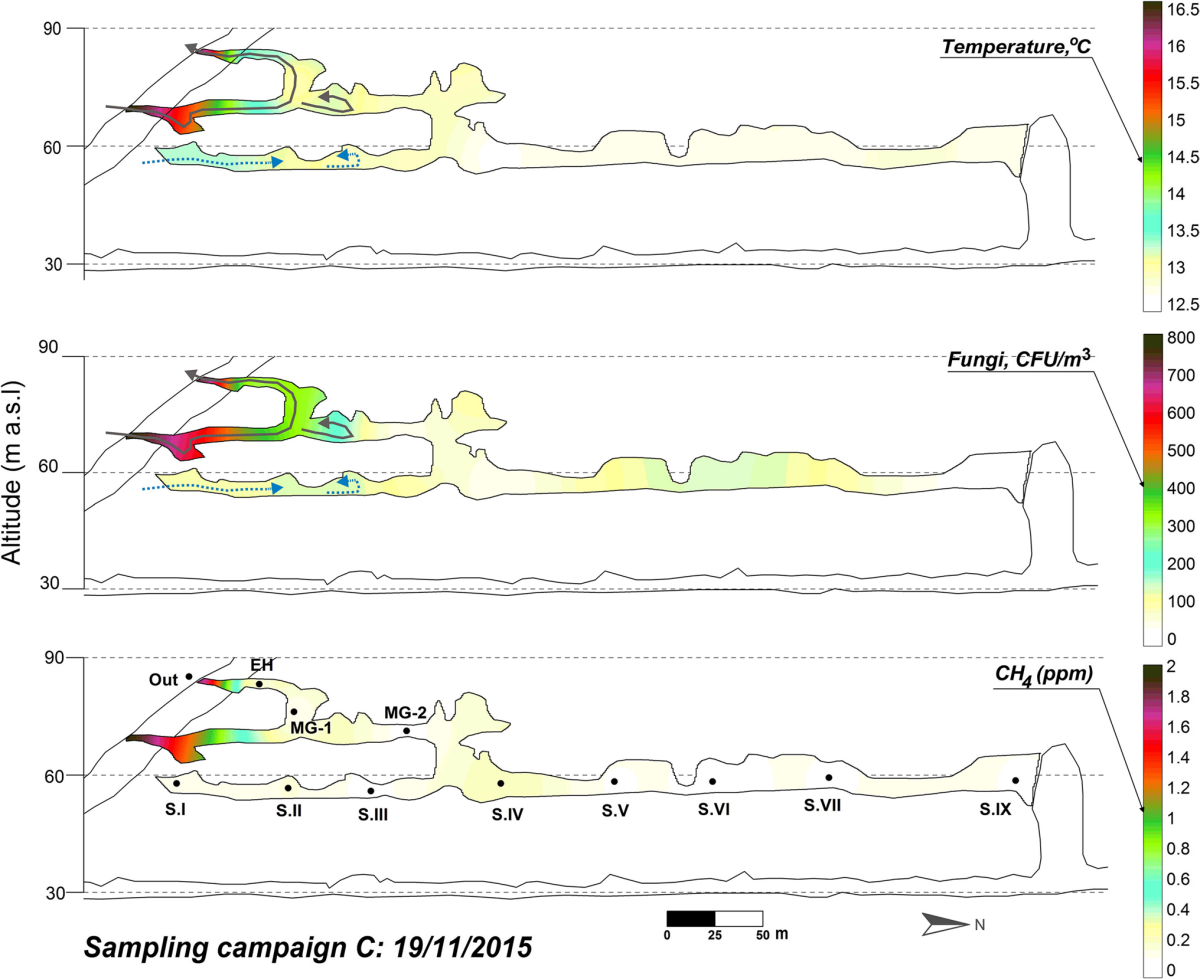
Spatial distribution of the fungal spore concentration, air temperature, and CH_4_ content across La Garma Cave in November 2015

During the air stagnation period of La Garma Cave (May–November), the air temperature in the Upper Gallery remains below the temperature in the rest of the lower galleries with a general pattern of thermal stability and absence of significant movements of air masses in the Lower Gallery. Under these climatic conditions, the levels of CH_4_ and fungal spores in the cave reach minimal concentrations compared to the rest of the samplings (November 2015, Table 3 and Fig. 8).

During this period, it was remarkable the practically total absence of the *Basidiomycota* observed in the ventilation period, excepting the finding of a few spores of *T. versicolor* and *C. micaceus* in the Sector IX, which could be a vestige from the ventilation period. Contrasting, *Ascomycota* were relatively abundant.

**Fig. 9:**
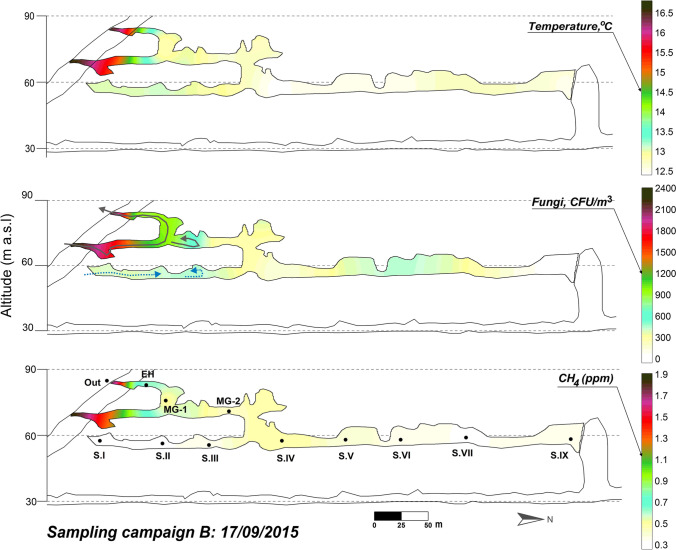
Spatial distribution of the fungal spore concentration, air temperature, and CH_4_ content across La Garma Cave in September 2015

At the end of summer and beginning of autumn, the instability outdoor intensifies and there are strong rises in the external temperature associated with periods of southern winds of notable intensity. In response, the cavity enters a period with abrupt variations of temperature at the upper levels and significant ventilation pulses mainly affecting the Upper and the Middle Galleries only in their areas closest to the entrance. These aerodynamic alterations have a minor impact on the environmental conditions of Sectors I and IV of the Lower Gallery and practically nil in Sector IX (Fig. 8). This period is characterized by the entry of fungal spores in the cave as noticed by the presence of *Pseudosubramaniomyces fusisaprophyticus*. This fungus is foreign to the cave and comes from outdoor, as evidenced in September 2015 sampling with their abundances outdoor and in the Entrance Hall, and the scarce abundance in the Middle Gallery (Figs. 5 and 9), not having been found in the rest of the cave sectors, neither in the November 2015 sampling. *Pseudosubramaniomyces fusisaprophyticus* is frequently found on fallen leaves, and especially from oak trees, in the early decomposition stage (Shirouzu et al. 2009), and is considered a typical litter colonizer (Shanthi and Vittal 2010). This agrees with the fact that the cave area was populated by *Quercus* spp*.*

*Beauveria* is a ubiquitous entomopathogenic genus (De Hoog 1972). This genus has also been recorded in a summer sampling in Ardales Cave in Málaga (Domínguez-Moñino 2015) and in Lascaux Cave (Bastian et al. 2009). *Beauveria varroae* was identified in September 2015 in Sector I (86% abundance), which would correspond to the population of insects that explodes and then dies in this season (Supplementary Table 2). This species is difficult to distinguish morphologically from *Beauveria bassiana*, a cosmopolitan species isolated from insects, soils, and caves (Nováková 2009; Rehner et al. 2011).

The genus *Lecanicillium* is composed of entomopathogenic species, which have previously been found in other caves (Nováková 2009; Vaughan et al. 2011). In La Garma, together with the species *Lecanicillium saksenae*, which appeared in the Entrance Hall in February 2016, *Lecanicillium aphanocladii* was found in September 2015 in the Entrance Hall and Sector IV, in the latter with an abundance of about 81% (Supplementary Table 2). The high incidence of entomopathogenic fungi in September 2015 is notable, since to *Lecanicillium aphanocladii* it must be added the presence of other entomopathogenic fungi such as *Cladosporium cladosporioides*, *Penicillium thomii*, and *Beauveria varroae* (Supplementary Table 2), *Purpureocillium lilacinum* (Supplementary Table 1), and *Parengyodontium album* (Supplementary Table 3). In September 2015, the entomopathogenic species represented a total of 16% in the Entrance Hall, 85% in Sector IV, and 86% in Sector I. This abundance is related to the population of insects that occurs in summer and their death at the end of summer. Some of these species have been frequently isolated in caves (Jurado et al. 2008; Bastian et al. 2009; Nováková 2009; Taylor et al. 2013; Domínguez-Moñino 2015; Leplat et al. 2020).

Related with arthropods is also the genus *Acrodontium*. Species of *Acrodontium* have been found on cave arthropods (Vanderwolf et al. 2019), particularly *A. salmoneum* on mites (Kubátová et al. 2001), whereas *A. luzulae* was isolated from dead leaves (Videira et al. 2016).

*Hypocrea lixii* was found in the Middle Gallery in September 2015 and also in the outdoor air in March 2015. The teleomorph *Hypocrea lixii* (= *Trichoderma lixii* and previously *Trichoderma harzianum*) is a cosmopolitan species known from all continents except Antarctica (Chaverri et al. 2003) and is common in humid forests and woods. Previously, this fungus was also isolated from other caves (Nováková 2009; Jurado et al. 2010). Therefore, the presence of *H. lixii* in the cave had a clear external origin and could be explained by ventilation pulses that reached the Middle Gallery.

Other interesting yeasts found inside La Garma Cave in September 15 with low abundances were *Tilletiopsis* spp. and *Sporobolomyces ruberrimus* (Supplementary Table 2). These species are commonly found on plant leaves and soils. The appearance of yeasts in nature takes place at the end of summer; hence, it was found in September 2015 in the cave air. Martin-Sanchez et al. (2014) sampled Lascaux Cave air in February and September and recorded species of the genera *Sporobolomyces*, *Cryptococcus*, and *Bulleromyces* only in September. In a further study, Martin-Sanchez and Saiz-Jimenez (2014) detected *Tilletiopsis pallescens*, reclassified as *Golubevia pallescens* (Richter et al. 2019), in the air of the same cave.

The genus *Aspergillus*, one of the most abundant in caves (Nováková 2009; Docampo et al. 2011), was scarcely represented in La Garma (Fig. 5). The highest abundances occurred in November 2015 for *A. versicolor* (17% in Sector IV) and September 2015 (7% in Sector I). Lower abundances were recorded in February 2016 for *A. ustus* (2.6% in Sector IX) and *A. ochraceus* (0.6% in the Entrance Hall). Outdoor was retrieved *A. sydowii* (March 2015), absent in the cave air.

During the period of greatest stagnation (November 2015) was noticeable the outdoor abundance of *C. cladosporioides*. The genus *Cladosporium* is composed of species common in nature and represents one of the genera with the largest number of species, with spores very abundant in the air and indoor environments (Bensch et al. 2018). However, their presence inside the cave was negligible in November 2015 and with some abundance in the Entrance Hall in September 2015. These abundances and those of *Ps. fusisaprophyticus* proved that the cave was relatively isolated from the exterior, and only the Entrance Hall suffered some impact from the exterior air.

Other interesting fungi detected inside the cave in the stagnation period were *Trichophyton terrestre* and *Pseudogymnoascus pannorum*. This last species, *C. cladosporioides* and *P. chrysogenum*, among other fungi, have been found on bat cadavers in a Slovakian cave (Nováková et al. 2018).

The genus *Trichophyton* is composed of a series of dermatophyte specie**s**. *Trichophyton terrestre* (Durie and Frey 1957) is a universally distributed saprophytic ascomycete, found on the skin of animals. This species is common in soils (geophile) and it was found in Sectors I (90% abundance) and IV (17% abundance) in November 2015**.** A *Trichophyton* sp. was also abundant in the air of the Painted Gallery, in Lascaux Cave (Martin-Sanchez et al. 2014).

The genus *Pseudogymnoascus* is notable for the outbreak of mortality among bats that inhabit the caves of North America. Initially described as produced by the fungus *Geomyces destructans*, it was reclassified as *Pseudogymnoascus destructans* (Minnis and Lindner 2013). The species found in La Garma Cave, *P. pannorum*, was found only in November 2015 in the Middle Gallery (33%) and Sector IV (50%) (Fig. 5, Supplementary Table 3). This species is relatively common in caves (Bastian et al. 2009; Nováková 2009; Martin-Sanchez et al. 2014) and was isolated from bats (Vanderwolf et al. 2013; Nováková et al. 2018). Their presence could be related to the hibernation of bats in the cave during the sampling period.

The genus *Auxarthronopsis* encompasses six species, from which five were isolated from samples of soils, rocks, feces, and plant debris collected in Chinese caves (Zhang et al. 2017, 2021). The genus was erected after the isolation of *Auxarthronopsis bandhavgarhensis* from an Indian soil (Sharma et al. 2013). A 50% abundance of *Auxarthronopsis* sp. was found in Sector IX (November, 2015). No further strains of this fungus were previously reported in caves.

Other *Ascomycota* that appear in the cave, with little abundance, are *Diaporthe* sp., *Stagonospora* sp., *Alternaria* spp., and *Leptosphaeria* sp., all plant pathogens. Its origin inside the cave may be due to the vegetation found in the proximity of the cavity, as suggested by its presence outdoor. Various species of the genus *Alternaria* were previously identified in caves (Bastian et al. 2009; Nováková 2009; Docampo et al. 2011; Domínguez-Moñino 2015).

### Airborne fungi and cave conservation

Air circulation is important for the transport and dispersion of airborne *Basidiomycota*, from outside to inside a cave. Most airborne fungi come from the external air that penetrates caves at entrances, as has been reported in this work. Other studies also support this statement. For instance, in Altamira Cave, the analysis of *Cladosporium* spp. and *Epicoccum nigrum* sequences from spores collected outdoor and inside the cave showed to be identical. This confirmed that the presence of spores of these two genera in the air of Altamira Cave was due to transport from the exterior by wind currents and ventilation (Garcia-Anton et al. 2014).

Archaeological activities, frequent inside the cave, can contribute to enrichments in carbon and nitrogen sources and enhance the colonization and growth of cave microorganisms. According to Jans et al. (2004), the majority of archaeological bones buried as fragments (e.g., from slaughtered animals) show fungal attacks. Fungal disturbance appears if environmental conditions are favorable for saprophytic fungi and can occur at any time, either during burial or after excavation.

A feature that could be of importance for the conservation of the cave and its archaeological remains is the ecology of *Trametes versicolor*. The growth and penetration of *T. versicolor* hyphae on apatite (a component of bones, of which it constitutes 70%) and the ability to produce corrosion on calcium phosphate were reported by Adeyeni and Gadd (2005). Pinzari et al. (2013) revealed the attack of ivory buried in the soil by *Ascomycota* and *Basidiomycota*. The deterioration increased in the presence of a carbon source. Child (1995) isolated *Penicillium chrysogenum*, *Penicillium expansum*, and *Cladosporium cladosporioides* from archaeological bones, which were also isolated in the cave. A previous report showed that the five intact Visigothic skeletons found in the Lower Gallery, dating to the seventh-eighth centuries AD, were colonized by fungi, likely *Penicillium* spp. (Camarós et al. 2021).

Therefore, it is suggested that the archaeological bones in La Garma Cave could be affected by airborne fungal spores coming from outdoor and deposited on the bones, which can lead to colonization and growth of fungi on bones, favored by the environmental humidity and the nutrients contained in the sediments. Alternatively, the fungi inhabiting bones can release their spores to the air.

Controlling airborne fungi in caves is a major challenge for microbiologists and curators because an appropriate method to avoid fungal spore dispersion and speleothem and archaeological bone colonization is a must. The use of non-invasive methods for the study and conservation of cultural heritage has gained increasing interest in recent, particularly those applied in subterranean sites (Pusz et al. 2015; Bercea et al. 2018; Leplat et al. 2019, 2020). Through the atmospheric analysis of both biological and gaseous composition of cave air, it is possible to provide important indications for interventions of prevention, conservation, and restoration of cultural heritage in these indoor environments. Any preventive conservation action must be adopted by evaluating periodically, through aerobiological and gas monitoring, the presence or risk of a fungal outbreak due to changes in cave aerodynamical conditions.

La Garma Cave is mainly affected by the transport of outdoor fungi that influences the indoor environment in terms of airborne composition. Human-induced impacts in this cave are controlled due to the closure of the cave to visits, and it is only affected by archaeological excavations and climate-driven ventilation. Corrective or preventive conservation measures in this cave should aim at not modifying and/or improve the current situation. Any change can provoke the proliferation of other fungal spores whose dispersal capacity toward the internal environment is unknown and, in any case, different from that characterized in this study under conditions very close to the natural ones. Archaeological excavation at the cave entrance should be limited or, at least, it must be deployed under strict measures aiming to prevent the movement and dispersion of airborne particles (e.g., by installing temporarily aerodynamic barriers at the entrances during the archaeological works). The archaeological intervention in the interior involving earth removal is inadvisable. The air stagnation stage during summer is the most suitable period for any archaeological intervention outdoor, but weather forecasting is essential to avoid the archaeological fieldwork, particularly in La Garma A Entrance, during the cave ventilation pulses due to the southern winds.

## Conclusions

The aerodynamic of La Garma Cave presents a great micro-environmental complexity due to the presence of several levels of galleries interconnected each other, as well as with the active subterranean river and the external atmosphere. The pattern results in lower temperatures in the Upper Gallery (Entrance Hall) and Middle Gallery than those of the Lower Gallery. This phenomenon of thermal inversion (less dense hot air below more dense cold air) implies certain aerodynamic instability and indicates that the entry of cold air, typical of the winter ventilation period, prevails over summer insulation in intensity and duration. The winter ventilation had an enormous influence on the dispersal of airborne fungi and the increasing spore concentrations in the cave air. The stagnation-ventilation regime inside the cave has a definite effect on the dispersion of fungal spores in La Garma Cave.

Considering the four samplings, in the cave diagram (Fig. 5), it is observed that the highest concentrations of fungi are found at the entrances of the Upper and Middle Galleries, showing decreasing values ​as one progress toward the Sectors I and IX. However, the Middle Gallery and its connection with the Lower Gallery (Sector IV) are also points of a great concentration of fungal spores.

In September–November, *Ascomycota* proliferated throughout the cave, while in February–March, they were replaced by *Basidiomycota*. This, together with the abundance of entomopathogenic fungi in September, indicates a marked seasonality in the behavior of airborne fungi.

Individualizing by cave periods of stagnation and ventilation, the cave reflects the seasonal changes of fungi and bats. Thus, the basidiomycete *Trametes versicolor* proliferated in the air when their spores were released to the atmosphere, while *Trichophyton terrestre*, *Cryptococcus* spp., and *Pseudogymnoascus pannorum*, related to the skin of animals, were found in the hibernation period of bats. The presence of *Beauveria varroae*, *Lecanicillium aphanocladii*, and other entomopathogenic fungi would correspond to the death of insects at the end of summer. Likewise, yeasts appeared in nature at the end of summer; hence, it was found in the cave in September. In summary, the microclimate and aerobiological studies point to a marked seasonality of cave fungi, being influenced by ventilation-stagnation processes, and the arthropods and animals visiting and/or inhabiting the cave.

## Supplementary Information

Below is the link to the electronic supplementary material.

Supplementary file1 (DOC 241 KB)

## Data Availability

Sequences were deposited into the GenBank database under accession numbers MW826125-MW826214. All other data available in Supplementary Material.

## References

[CR1] Abrego N, García-Baquero G, Halme P, Ovaskainen O, Salcedo I (2014) Community turnover of wood-inhabiting fungi across hierarchical spatial scales. PLoS One 9: e103416 10.1371/journal.pone.0103416 PMC4110023 25058128

[CR2] Adeyeni AO, Gadd GM (2005). Fungal degradation of calcium-, lead- and silicon-bearing minerals. Biometals.

[CR3] Alabouvette C, Saiz-Jimenez C (2016). Écologie microbienne de la Grotte de Lascaux. Notes Acad Agric France.

[CR4] Alonso L, Pommier T, Kaufmann B, Dubost A, Chapulliot D, Doré J, Douady CJ, Moënne-Loccoz Y (2019). Anthropization level of Lascaux Cave microbiome shown by regional-scale comparisons of pristine and anthropized caves. Mol Ecol.

[CR5] Andersson H (2004). Pilze in der Einhornhöhle bei Scharzfeld (Harz/Niedersachsen). Mitt Verb Dt Höhlen- u Karstforscher.

[CR6] Arias P, Ontañón R, Bergsvik KA, Skeates R (2012). La Garma (Spain): Long-term human activity in a karstic system. Caves in context.

[CR7] Arias P, Ontañón R, Sala R (2014). La Garma. Los cazadores recolectores del Pleistoceno y del Holoceno en Iberia y el Estrecho de Gibraltar.

[CR8] Barr TC (1968) Ecological studies in the Mammoth Cave System of Kentucky. I. The Biota. Int J Speleol 3:147–204

[CR9] Bastian F, Alabouvette C, Saiz-Jimenez C (2009). The impact of arthropods on fungal community structure in Lascaux Cave. J Appl Microbiol.

[CR10] Bastian F, Jurado V, Nováková A, Alabouvette C, Saiz-Jimenez C (2010). The microbiology of the Lascaux Cave. Microbiology.

[CR11] Bensch K, Groenewald JZ, Meijer M, Dijksterhui J, Jurjevic Z, Andersen B, Houbraken J, Crous PW, Samson RA (2018). *Cladosporium* species in indoor environments. Stud Mycol.

[CR12] Bercea S, Nastase-Bucur R, Mirea IC, Mantoiu DS, Kenesz M, Petculescu A, Baricz A, Andrei A-S, Banciu HL, Papp B, Constantin S, Moldovan OT (2018). ﻿Novel approach to microbiological air monitoring in show caves. Aerobiologia.

[CR13] Call RE (1897). Some notes on the flora and fauna of Mammoth Cave, Ky. Am Nat.

[CR14] Camarós E, López-Gijón R, Zurro D, Gutiérrez-Zugasti I, Sánchez-Moral S, Martín-Pozas T, Miguel Sánchez Carro MA, Gleba M, Ontañón R, Arias P (2021) Microtaphonomic approach to archaeothanatology: a crossdisciplinary perspective from a Visigothic funerary cave context (La Garma, Cantabrian Spain). 27th European Association of Archaeologists, Abstract 1450

[CR15] Chaverri P, Castlebury LA, Samuels GJ, Geiser DM (2003). Multilocus phylogenetic structure within the *Trichoderma harzianum*/*Hypocrea lixii* complex. Mol Phylogen Evol.

[CR16] Child AM (1995). Towards an understanding of the microbial decomposition of archaeological bone in the burial environment. J Archaeol Sci.

[CR17] Cigna AA (2016) Tourism and show caves. Ann Geomorphol 60:217–233

[CR18] Comas-Bru L, McDermott F (2015). Data-model comparison of soil-water δ^18^O at a temperate site in N. Spain with implications for interpreting speleothem δ^18^O. J Hydrol.

[CR19] Cuezva S, Fernández-Cortés A, Jurado V, Sáiz-Jiménez C, Ontañón R, Arias P, Hernández-Vicente I, Sanchez-Moral S, Andreo B, Durán JJ (2016). Investigación aplicada a la conservación preventiva del sistema kárstico de la Garma (Omoño, Ribamontan al Monte, Cantabria). El Karst y el hombre: las cuevas como patrimonio mundial.

[CR20] Cuezva S, Fernandez-Cortes A, Benavente D, Serrano-Ortiz P, Kowalski AS, Sanchez-Moral S (2011). Short-term CO_2_(g) exchange between a shallow karstic cavity and the external atmosphere during summer: Role of the surface soil layer. Atmos Environ.

[CR21] De Hoog GS (1972). The genera *Beauveria, Isaria, Tritirachium* and *Acrodontium* gen. nov. Stud Mycol.

[CR22] Docampo S, Trigo MM, Recio M, Melgar M, García-Sánchez J, Cabezudo B (2011). Fungal spores content of the atmosphere of the Cave of Nerja (southern Spain): Diversity and origin. Sci Total Environ.

[CR23] Domínguez-Moñino I (2015). Evaluación y control de comunidades microbianas en cuevas turísticas.

[CR24] Domínguez-Moñino I, Jurado V, Hermosín B, Sáiz-Jiménez C, Durán JJ, Robledo PA (2012). Aerobiología de cuevas andaluzas. Las cuevas turísticas como activos económicos: Conservación e innovación.

[CR25] Domínguez-Moñino I, Jurado V, Hermosín B, Sáiz-Jiménez C, Calaforra JM, Durán JJ (2014). Aerobiología de la Gruta de las Maravillas. Cuevatur 2014/Iberoamérica Subterránea.

[CR26] Dominguez-Moñino I, Jurado V, Rogerio-Candelera MA, Hermosin B, Saiz-Jimenez C (2021). Airborne fungi in show caves from Southern Spain. Appl Sci.

[CR27] Durie EB, Frey D (1957). A new species of *Trichophyton* from New South Wales. Mycologia.

[CR28] Fernandez-Cortes A, Cuezva S, Sanchez-Moral S, Porca E, Jurado V, Saiz-Jimenez C (2011). Detection of human-induced environmental disturbances in a show cave. Environ Sci Pollut Res.

[CR29] Fernandez-Cortes A, Cuezva S, Garcia-Anton E, Alvarez-Gallego M, Pla C, Benavente D, Cañaveras JC, Calaforra JM, Mattey DP, Sanchez-Moral S (2015). Changes in the storage and sink of carbon dioxide in subsurface atmospheres controlled by climate-driven processes: the case of the Ojo Guareña karst system. Environ Earth Sci.

[CR30] Fernandez-Cortes A, Cuezva S, Alvarez-Gallego M, Garcia-Anton E, Pla C, Benavente D, Jurado V, Saiz-Jimenez C, Sanchez-Moral S (2015). Subterranean atmospheres may act as daily methane sink. Nature Comm.

[CR31] García-Anton E, Cuezva S, Jurado V, Porca E, Miller AZ, Fernandez-Cortes A, Saiz-Jimenez C, Sanchez-Moral S (2014). Combining stable isotope (δ^13^C) of trace gases and aerobiological data to monitor the entry and dispersion of microorganisms in caves. Environ Sci Pollut Res.

[CR32] Garcia-Anton E, Cuezva S, Fernadez-Cortes A, Alvarez-Gallego M, Pla C, Benavente D, Cañaveras JC, Sanchez-Moral S (2017). Abiotic and seasonal control of soil-produced CO_2_ efflux in karstic ecosystems located in Oceanic and Mediterranean climates. Atmos Environ.

[CR33] Geneste J-M, Mauriac M, Saiz-Jimenez C (2014). The conservation of Lascaux Cave, France. The conservation of subterranean cultural heritage.

[CR34] Gottstein Matocec S (2001). An overview of the cave and interstitial biota of Croatia. Nat Croat.

[CR35] Hoffmann A, Palacios-Vega JG, Morales-Malacara JB (1986). Manual de Bioespeleología.

[CR36] Jans MME, Nielsen-Marsh CM, Smith CI, Collins MJ, Kars H (2004). Characterisation of microbial attack on archaeological bone. J Archaeol Sci.

[CR37] Jurado V, Sanchez-Moral S, Saiz-Jimenez C (2008). Entomogenous fungi and the conservation of the cultural heritage: a review. Int Biodeter Biodegr.

[CR38] Jurado V, Fernandez-Cortes A, Cuezva S, Laiz L, Cañaveras JC, Sanchez-Moral S, Saiz-Jimenez C (2009). The fungal colonization of rock art caves. Naturwissenschaften.

[CR39] Jurado V, Porca E, Cuezva S, Fernandez-Cortes A, Sanchez-Moral S, Saiz-Jimenez C (2010). Fungal outbreak in a show cave. Sci Total Environ.

[CR40] Justo A, Hibbett DS (2011). Phylogenetic classification of *Trametes* (Basidiomycota, Polyporales) based on a five-marker dataset. Taxon.

[CR41] Kubátová A, Cerný M, Nováková A (2001). New records of micromycetes from the Czech Republic. IV. Acrodontium salmoneum, Chaunopycnis alba and Cylindrocarpostylus gregarius, and notes on Dactylaria lanosa and Trichoderma saturnisporum. Czech Mycol.

[CR42] Lasheras JA, de las Heras C, Prada A, , Saiz-Jimenez C (2014). Altamira and its future. The conservation of subterranean cultural heritage.

[CR43] Leplat J, François A, Touron S, Galant P, Bousta F (2019). Aerobiological behavior of Paleolithic decorated caves: a comparative study of five caves in the Gard department (France). Aerobiologia.

[CR44] Leplat J, François A, Bousta F (2020). *Parengyodontium album*, a frequently reported fungal species in the cultural heritage environment. Fungal Biol Rev.

[CR45] Martin-Sanchez PM, Saiz-Jimenez C, Saiz-Jimenez C (2014). Contribution of culture-independent methods to cave aerobiology: the case of Lascaux Cave. The conservation of subterranean cultural heritage.

[CR46] Martin-Sanchez PM, Nováková A, Bastian F, Alabouvette C, Saiz-Jimenez C (2012a) Two new species of the genus *Ochroconis*, *O. lascauxensis* and *O. anomala* isolated from black stains in Lascaux Cave, France. Fungal Biol 116:574–589 10.1016/j.funbio.2012.02.006 22559918

[CR47] Martin-Sanchez PM, Nováková A, Bastian F, Alabouvette C, Saiz-Jimenez C (2012). Use of biocides for the control of fungal outbreaks in subterranean environments: the case of the Lascaux Cave in France. Environ Sci Technol.

[CR48] Martin-Sanchez PM, Jurado V, Porca E, Bastian F, Lacanette D, Alabouvette C, Saiz-Jimenez C (2014). Airborne microorganisms in Lascaux Cave (France). Int J Speleol.

[CR49] Martin-Sanchez P, Miller AZ, Saiz-Jimenez C, Engel AS (2015). Lascaux Cave: an example of fragile ecological balance in subterranean environments. Microbial life of cave systems.

[CR50] Mattey DP, Fisher R, Atkinson TC, Latin JP, Durrell R, Ainsworth M, Lowry D, Fairchild IJ (2013). Methane in underground air in Gibraltar karst. Earth Planet Sci Lett.

[CR51] Minnis AM, Lindner DL (2013). Phylogenetic evaluation of *Geomyces* and allies reveals no close relatives of *Pseudogymnoascus destructans*, comb. nov., in bat hibernacula of eastern North America. Fungal Biol.

[CR52] Motato-Vásquez V, Gugliotta AM, Rajchenberg M, Catania M, Urcelay C, Robledo G (2020). New insights on *Bjerkandera* (Phanerochaetaceae, Polyporales) in the Neotropics with description of *Bjerkandera albocinerea* based on morphological and molecular evidence. Plant Ecol Evol.

[CR53] Mulec J, Oarga-Mulec A, Šturm S, Tomazin R, Matos T (2017). Spacio-temporal distribution and tourist impact on airborne bacteria in a cave (Škocjan Caves, Slovenia). Diversity.

[CR54] Mulec J, Simčič S, Kotar T, Kofol R, Stopinšek S (2020). Survey of *Histoplasma capsulatum* in bat guano and status of histoplasmosis in Slovenia, Central Europe. Int J Speleol.

[CR55] Northup DE, Lavoie KH (2001). Geomicrobiology of caves: a review. Geomicrobiol J.

[CR56] Nováková A (2009). Microscopic fungi isolated from the Domica Cave system (Slovak Karst National Park, Slovakia), a review. Int J Speleol.

[CR57] Nováková A, Hubka V, Saiz-Jimenez C, Kolarik M (2012). *Aspergillus baeticus* sp. nov. and *Aspergillus thesauricus* sp. nov.: two new species in section Usti originating from Spanish caves. Int J Syst Evol Microbiol.

[CR58] Novakova A, Jurado V, Saiz-Jimenez C, Saiz-Jimenez C (2014). Are fungi a real threat for the conservation of Altamira Cave?. The conservation of subterranean cultural heritage.

[CR59] Novakova A, Hubka V, Saiz-Jimenez C, Saiz-Jimenez C (2014). Microscopic fungi isolated from cave air and sediments in the Nerja Cave-preliminary results. The conservation of subterranean cultural heritage.

[CR60] Nováková A, Kubátová A, Sklenář F, Hubka V (2018). Microscopic fungi on cadavers and skeletons from cave and mine environments. Czech Mycol.

[CR61] Ogórek R (2018). Speleomycology of air in Demänovská Cave of Liberty (Slovakia) and new airborne species for fungal sites. J Cave Karst Stud.

[CR62] Ojeda L, Vadillo I, Etiope G, Benavente J, Liñán C, del Rosal Y, Tapia ST, Morínigo MA, Carrasco F (2019). Methane sources and sinks in karst systems: the Nerja cave and its vadose environment (Spain). Geochim Cosmochim Acta.

[CR63] Ontañón R (2003). Sols et structures d’habitat du Paléolithique supérieur, nouvelles données depuis les Cantabres: La Galerie Inférieure de La Garma (Cantabrie, Espagne). L’anthropologie.

[CR64] Pinzari F, Tate J, Bicchieri M, Rhee YJ, Gadd GM (2013). Biodegradation of ivory (natural apatite): possible involvement of fungal activity in biodeterioration of Lewis Chessmen. Environ Microbiol.

[CR65] Pla C, Cuezva S, Martinez-Martinez J, Fernandez-Cortes A, Garcia-Anton E, Fusi N, Crosta GB, Cuevas-Gonzalez J, Cañaveras JC, Sanchez-Moral S, Benavente D (2017). Role of soil pore structure in water infiltration and CO_2_ exchange between the atmosphere and underground air in the vadose zone: A combined laboratory and field approach. Catena.

[CR66] Porca E, Jurado V, Martin-Sanchez PM, Hermosin B, Bastian F, Alabouvette C, Saiz-Jimenez C (2011). Aerobiology: an ecological indicator for early detection and control of fungal outbreaks in caves. Ecol Indic.

[CR67] Pusz W, Ogórek R, Knapik R, Kozak B, Bujak H (2015) The occurrence of fungi in the recently discovered Jarkowicka Cave in the Karkonosze Mts. (Poland). Geomicrobiol J 32: 59–67

[CR68] Rehner SA, Minnis AM, Sung G-H, Luangsa-ard JJ, Devotto L, Humber RA (2011). Phylogeny and systematics of the anamorphic, entomopathogenic genus *Beauveria*. Mycologia.

[CR69] Richter C, Yurkov AM, Boekhout T, Stadler M (2019). Diversity of *Tilletiopsis*-like fungi in Exobasidiomycetes (Ustilaginomycotina) and description of six novel species. Front Microbiol.

[CR70] Saiz-Jimenez C, Cuezva S, Jurado V, Fernandez-Cortes A, Porca E, Benavente D, Cañaveras JC, Sanchez-Moral S (2011). Paleolithic art in peril: policy and science collide at Altamira Cave. Science.

[CR71] Saiz-Jimenez C (2013) Cave conservation: a microbiologist’s perspective. In: Chhepham N (ed) Cave microbiomes: a novel resource for drug discovery. SpringerBrief in Microbiology 1, New York, pp- 69–84

[CR72] Shanthi S, Vittal BPR (2010). Fungi associated with decomposing leaf litter of cashew (*Anacardium occidentale*). Mycology.

[CR73] Sharma R, Gräser Y, Singh SK (2013). *Auxarthronopsis*, a new genus of *Onygenales* isolated from the vicinity of Bandhavgarh National Park, India. IMA Fungus.

[CR74] Shirouzu T, Hirose D, Fukasawa Y, Tokumasu S (2009). Fungal succession associated with the decay of leaves of an evergreen oak, *Quercus myrsinaefolia*. Fungal Divers.

[CR75] Sugita T, Kikuchi K, Makimura K, Urata K, Someya T, Kamei K, Niimi M, Uehara Y (2005). *Trichosporon* species isolated from guano samples obtained from bat-inshabited caves in Japan. Appl Environ Microbiol.

[CR76] Taylor ELS, Resende-Stoianoff MAA, Lopes Ferreira R (2013). Mycological study for a management plan of a neotropical show cave (Brazil). Int J Speleol.

[CR77] Telleria MT, Dueñas M, Melo I, Martín M (2010). Morphological and molecular studies of *Hyphodermella* in the Western Mediterranean area. Mycol Progress.

[CR78] Vanderwolf K, McAlpine DF, Malloch D, Forbes GJ (2013). Ectomycota associated with hibernating bats in eastern Canadian caves prior to the emergence of white-nose syndrome. Northeast Nat.

[CR79] Vanderwolf K, Malloch D, McAlpine DF (2019). No change detected in culturable fungal assemblages on cave walls in Eastern Canada with the introduction of *Pseudogymnoascus destructans*. Diversity.

[CR80] Vaughan MJ, Maier RM, Pryor BM (2011). Fungal communities on speleothem surfaces in Kartchner Caverns, Arizona, USA. Int J Speleol.

[CR81] Vaughan-Martini A, Angelini P, Zacchi L (2000). The influence of human and animal visitation on the yeast ecology of three Italian caverns. Ann Microbiol.

[CR82] Videira SIR, Groenewald JZ, Braun U, Shin HD, Crous PW (2016). All that glitters is not *Ramularia*. Stud Mycol.

[CR83] Visagie CM, Houbraken J, Frisvad JC, Hong S-B, Klaassen CHW, Perrone G, Seifert KA, Varga J, Yaguchi T, Samson RA (2014). Identification and nomenclature of the genus *Penicillium*. Stud Mycol.

[CR84] Waring CL, Hankin SI, Griffith DWT, Kertesz MA, Kobylski V, Wilson NL, Coleman NV, Kettlewell G, Zlot R, Bosse M, Bell G (2017). Seasonal total methane depletion in limestone caves. Sci Rep.

[CR85] Welden AL (1971). An essay on *Stereum*. Mycologia.

[CR86] Webster KD, Drobniak A, Etiope G, Mastalerz M, Sauer PE, Schimmelmann A (2018). Subterranean karst environments as a global sink for atmospheric methane. Earth Planet Sci Lett.

[CR87] White TJ, Bruns T, Lee S, Taylor J, Innis MA, Gelfand DH, Sninsky JJ, White TJ (1990). Amplification and direct sequencing of fungal ribosomal RNA genes for phylogenetics. PCR protocols: a guide to methods and applications.

[CR88] Wojkowski J, Andreychouk V, Frączek K (2019). Airborne microorganisms of hypogenic maze caves based on the example of the Zoloushka Cave, Ukraine-Moldova. Rocz Ochr Środowiska.

[CR89] Zhang T, Victor TR, Rajkumar SS, Li X, Okoniewski JC, Hicks AC, Davis AD, Broussard K, LaDeau SL, Chaturvedi S, Chaturvedi V (2014) Mycobiome of the bat white nose syndrome affected caves and mines reveals diversity of fungi and local adaptation by the fungal pathogen *Pseudogymnoascus* (*Geomyces*) *destructans*. PLoS One 9:e108714 10.1371/journal.pone.0108714 PMC4181696 25264864

[CR90] Zhang ZF, Liu F, Zhou X, Liu XZ, Liu SJ, Cai L (2017). Culturable mycobiota from Karst caves in China, with descriptions of 20 new species. Persoonia.

[CR91] Zhang ZF, Zhou SY, Eurwilaichitr L, Ingsriswang S, Raza M, Chen Q, Zhao P, Liu F, Cai L (2021) Culturable mycobiota from Karst caves in China II, with descriptions of 33 new species. Fungal Divers 106:29–136

