# Supplementary material for: Environment-driven control of fungi in subterranean ecosystems: the case of La Garma Cave (northern Spain)
Source: Int Microbiol. 2021 Jul 22;24(4):573–91. doi: 10.1007/s10123-021-00193-x (PMC8616876; doi:10.1007/s10123-021-00193-x)
Supplement: Supplementary file 1 — Supplementary file1 (DOC 241 KB) [file 10123_2021_193_MOESM1_ESM.doc]

**Supplementary Table 1.** Sampling campaign March 24, 2015. UFC/m^3^, accession numbers, identifications and abundances.

| **Sampling** | **UFC/m^3*^** | | **Accession Number** | **Identification (% identity)** | **Abundance (%)** |
| --- | --- | --- | --- | --- | --- |
| Entrance Hall | ND | |  |  |  |
| Middle Gallery | 5,420  (30) | | MW826209 | *Bjerkandera adusta* (100%) | 2.00 |
|  |  |  | MW826210 | *Hyphodermella rosae* (100%) | 0.05 |
|  |  |  | MW826207 | *Penicillium minioluteum* (100%) | 0.25 |
|  |  |  | MW826208 | *Stereum hirsutum* (100%) | 4.00 |
|  |  |  | MW826211 | *Trametes versicolor* (100%) | 93.25 |
| Sector IX | 640  (40) | | MW826201 | *Cladosporium cladosporioides* 100% | 1.79 |
|  |  |  | MW826212 | *Purpureocillium lilacinum* (100%) | 0.89 |
|  |  |  | MW826214 | *Phaeophlebiopsis peniophoroides* (100%) | 1.79 |
|  |  |  | MW826211 | *Trametes versicolor* (100%) | 88.39 |
|  |  |  | MW826213 | *Phanerochaete livescens* (100%) | 1.79 |
|  |  |  | MW826208 | *Stereum hirsutum* (100%) | 5.36 |
| Sector IV | 2,650  (80) | | MW826190 | *Penicillium digitatum* (100%) | 0.65 |
|  |  |  | MW826191 | *Penicillium diversum* (96%) | 0.33 |
|  |  |  | MW826208 | *Stereum hirsutum* (100%) | 43.97 |
|  |  |  | MW826210 | *Hyphodermella rosae* (100%) | 30.62 |
|  |  |  | MW826211 | *Trametes versicolor* (100%) | 24.43 |
| Sector I | 390  (10) | | MW826192 | *Alternaria chlamydosporigena* (99%) | 6.94 |
|  |  |  | MW826204 | *Alternaria infectoria* (100%) | 1.39 |
|  |  |  | MW826201 | *Cladosporium cladosporioides* 100% | 1.39 |
|  |  |  | MW826197 | *Crustomyces subabruptus* (99%) | 4.17 |
|  |  |  | MW826194 | *Cryptococcus stepposus* (99%) | 70.83 |
|  |  |  | MW826195 | *Naganishia diffluens* (100%) | 5.56 |
|  |  |  | MW826196 | *Phialemonium* aff. *dimorphosporum* (94%) | 6.94 |
|  |  |  | MW826193 | *Penicillium chrysogenum* (100%) | 2.78 |
| Outdoor | | 1,520  (40) | MW826206 | *Aspergillus sydowii* (100%) | 3.18 |
|  |  |  | MW826204 | *Alternaria infectoria* (100%) | 0.45 |
|  |  |  | MW826201 | *Cladosporium cladosporioides* (100%) | 2.27 |
|  |  |  | MW826200 | *Diaporthe foeniculina* (100%) | 1.83 |
|  |  |  | MW826203 | *Fusarium tricinctum* (99%) | 0.45 |
|  |  |  | MW826198 | *Hypocrea lixii* (99%) | 0.45 |
|  |  |  | MW826202 | *Leptosphaeria* sp. (100%) | 1.83 |
|  |  |  | MW826199 | *Pestalotiopsis microspora* (98%) | 0.91 |
|  |  |  | MW826208 | *Stereum hirsutum* (100%) | 6.36 |
|  |  |  | MW826205 | *Stereum sanguinolentum* (99%) | 36.36 |
|  |  |  | MW826211 | *Trametes versicolor* (100%) | 45.91 |

*****Data refer to average and standard deviation in brackets.

ND: Not Determined

**Supplementary Table 2.** Sampling campaign September 17, 2015. UFC/m^3^, accession numbers, identifications and abundances.

| **Sampling** | **UFC/m^3^** | **Accession Number** | **Identification (% identity)** | **Abundance (%)** |
| --- | --- | --- | --- | --- |
| Entrance Hall | 810  (40) | MW826157 | *Aspergillus versicolor* (100%) | 2.21 |
|  |  | MW826155 | *Pseudosubramaniomyces fusisaprophyticus* (94%) | 75.00 |
|  |  | MW826151 | *Cladosporium cladosporioides* (100%) | 8.82 |
|  |  | MW826150 | *Colletotrichum camelliae* (99%) | 5.15 |
|  |  | MW826143 | *Lecanicillium aphanocladii* (100%) | 1.47 |
|  |  | MW826156 | *Penicillium hispanicum* (100%) | 2.21 |
|  |  | MW826152 | *Penicillium thomii* (100%) | 0.74 |
|  |  | MW826158 | *Schizophyllum commune* (100%) | 4.41 |
| Middle Gallery | 230  (40) | MW826155 | *Pseudosubramaniomyces fusisaprophyticus* (94%) | 4.65 |
|  |  | MW826160 | *Diaporthe* sp. (100%) | 2.33 |
|  |  | MW826161 | *Epicoccum plurivorum* (100%) | 2.33 |
|  |  | MW826159 | *Hypocrea lixii* (99%) | 46.51 |
|  |  | MW826163 | *Penicillium chrysogenum* (100%) | 25.58 |
|  |  | MW826164 | *Penicillium brevicompactum* (100%) | 6.98 |
|  |  | MW826165 | *Penicillium westlingii* (98%) | 4.65 |
|  |  | MW826162 | *Phlebia acerina* (100%) | 6.98 |
| Sector  IX | 40  (10) | MW826168 | *Acrodontium salmoneum* (99%) | 25.00 |
|  |  | MW826169 | *Acrodontium luzulaei* (100%) | 12.50 |
|  |  | MW826166 | *Tilletiopsis washingtonensis* (99%) | 37.50 |
|  |  | MW826167 | *Trametes versicolor* (99%) | 12.50 |
|  |  | MW826170 | *Phaeophlebiopsis peniophoroides* (99%) | 12.50 |
| Sector IV | 130  (30) | MW826151 | *Cladosporium cladosporioides* (100%) | 3.85 |
|  |  | MW826143 | *Lecanicillium aphanocladii* (100%) | 80.77 |
|  |  | MW826144 | *Tilletiopsis pallescens* (100%) | 15.38 |
| Sector  I | 380  (60) | MW826145 | *Beauveria varroae* (100%) | 85.51 |
|  |  | MW826148 | *Aspergillus versicolor* (100%) | 7.25 |
|  |  | MW826149 | *Acrodontium crateriforme* (99%) | 2.90 |
|  |  | MW826146 | *Sporobolomyces ruberrrimus* (99%) | 2.90 |
|  |  | MW826147 | *Cystobasidium slooffiae* (99%) | 1.45 |
| Outdoor | 2,280  (150) | MW826155 | *Pseudosubramaniomyces fusisaprophyticus* (94%) | 90.46 |
|  |  | MW826150 | *Colletotrichum camelliae* (99%) | 1.41 |
|  |  | MW826151 | *Cladosporium cladosporioides* (100%) | 5.30 |
|  |  | MW826154 | *Diaporthe rudis* (99%) | 1.77 |
|  |  | MW826152 | *Penicillium thomii* (100%) | 0.35 |
|  |  | MW826153 | *Trichoderma* sp. (100%) | 0.71 |

**Supplementary Table 3.** Sampling campaign November 19, 2015. UFC/m^3^, accession numbers, identifications and abundances.

| **Sampling** | **UFC/m^3^** | **Accession Number** | **Identification (% identity)** | **Abundance (%)** |
| --- | --- | --- | --- | --- |
| Entrance Hall | ND |  |  |  |
| Middle Gallery | 30  (0) | MW826187 | *Cladosporium cladosporioides* (100%) | 16.67 |
|  |  | MW826186 | *Jalapriya pulchra* (100%) | 16.67 |
|  |  | MW826173 | *Pseudogymnoascus pannorum* (100%) | 33.33 |
|  |  | MW826185 | *Microascus paisii* (99%) | 33.33 |
| Sector  IX | 10  (0) | MW826188 | *Auxarthronopsis* sp. (99%)  *Coprinellus micaceus* (99%) | 50.00  50.00 |
|  |  | MW826189 |  |  |
| Sector  IV | 30  (10) | MW826171 | *Talaromyces rugulosus* (99%)  *Aspergillus versicolo*r (100%)  *Pseudogymnoascus pannorum* (100%) *Trichophyton terrestre* (99%) | 16.67  16.67  50.00  16.67 |
|  |  | MW826172 |  |  |
|  |  | MW826173 |  |  |
|  |  | MW826174 |  |  |
| Sector I | 110  (20) | MW826176 | *Parengyodontium album* (100%)  *Penicillium roqueforti* (100%)  *Trichophyton terrestre* (99%) | 4.76  4.76  90.48 |
|  |  | MW826175 |  |  |
|  |  | MW826174 |  |  |
| Outdoor | 780  (80) | MW826187 | *Cladosporium cladosporioides* (100%) | 71.00 |
|  |  | MW826177 | *Acrodontium salmoneum* (99%) | 4.59 |
|  |  | MW826179 | *Leptosphaeria* sp. (100%) | 1.53 |
|  |  | MW826178 | *Penicillium brevicompactum* (100%) | 4.58 |
|  |  | MW826180 | *Phoma macrostoma* (100%) | 2.29 |
|  |  | MW826181 | *Periconia* sp. (100%) | 5.34 |
|  |  | MW826183 | *Peniophora lycii* (100%) | 2.29 |
|  |  | MW826184 | *Phlebia acerina* (100%) | 1.52 |
|  |  | MW826182 | *Trametes versicolor* (100%) | 6.87 |

ND: Not Determined

**Supplementary Table 4.** Sampling campaign February 17, 2016. UFC/m^3^, accession numbers, identifications and abundances.

| **Sampling** | **UFC/m^3^** | **Accession Number** | **Identification (% identity)** | **Abundance (%)** |
| --- | --- | --- | --- | --- |
| Entrance Hall | 1,010  (80) | MW826133 | *Aspergillus ochraceus* (100%) | 0.62 |
|  |  | MW826128 | *Cladosporium cladosporioides* (100%) | 1.24 |
|  |  | MW826134 | *Coprinellus micaceus* (100%) | 95.03 |
|  |  | MW826131 | *Lecanicillium saksenae* (98%) | 0.62 |
|  |  | MW826138 | *Leptosphaeria* sp. (100%) | 0.62 |
|  |  | MW826129 | *Penicillium expansum* (100%) | 0.62 |
|  |  | MW826132 | *Penicillium miczynskii* (100%) | 0.62 |
|  |  | MW826135 | *Penicillium brevicompactum* (100%) | 0.62 |
| Middle Gallery | 320  (40) | MW826130 | *Bjerkandera adusta* (100%) | 81.36 |
|  |  | MW826138 | *Leptosphaeria* sp. (100%) | 5.08 |
|  |  | MW826136 | *Penicillium vancouverense* (100%) | 1.70 |
|  |  | MW826135 | *Penicillium brevicompactum* (100%) | 10.17 |
|  |  | MW826137 | *Stagonospora* sp. (100%) | 1.70 |
| Sector IX | 420  (40) | MW826140 | *Aspergillus ustus* (100%) | 2.63 |
|  |  | MW826128 | *Cladosporium cladosporioides* (100%) | 3.95 |
|  |  | MW826139 | *Penicillium aurantiovirens* (100%) | 1.32 |
|  |  | MW826141 | *Penicillium chrysogenum* (100%) | 21.05 |
|  |  | MW826127 | *Trametes versicolor* (99%) | 71.05 |
| Sector IV | 380  (30) | MW826141 | *Penicillium chrysogenum* (100%) | 1.43 |
|  |  | MW826135 | *Penicillium brevicompactum* (100%) | 1.43 |
|  |  | MW826126 | *Phanerochaete livescens* (100%) | 28.57 |
|  |  | MW826125 | *Stereum hirsutum* (100%) | 21.43 |
|  |  | MW826127 | *Trametes versicolor* (99%) | 47.14 |
| Sector I | 200  (10) | MW826141 | *Penicillium chrysogenum* (100%) | 23.68 |
|  |  | MW826139 | *Penicillium aurantiovirens* (100%) | 2.63 |
|  |  | MW826125 | *Stereum hirsutum* (100%) | 2.63 |
|  |  | MW826127 | *Trametes versicolor* (99%) | 71.05 |
| Outdoor | 150  (40) | MW826130 | *Bjerkandera adusta* (100%) | 47.22 |
|  |  | MW826128 | *Cladosporium cladosporioides* (100%) | 5.56 |
|  |  | MW826141 | *Penicillium chrysogenum* (100%) | 2.78 |
|  |  | MW826142 | *Penicillium freii* (100%) | 8.33 |
|  |  | MW826129 | *Penicillium expansum* (100%) | 2.78 |
|  |  | MW826125 | *Stereum hirsutum* (100%) | 22.22 |
|  |  | MW826127 | *Trametes versicolor* (99%) | 11.11 |
